# Serum protein fingerprinting by PEA immunoassay coupled with a pattern-recognition algorithms distinguishes MGUS and multiple myeloma

**DOI:** 10.18632/oncotarget.11242

**Published:** 2016-08-12

**Authors:** Petra Schneiderova, Tomas Pika, Petr Gajdos, Regina Fillerova, Pavel Kromer, Milos Kudelka, Jiri Minarik, Tomas Papajik, Vlastimil Scudla, Eva Kriegova

**Affiliations:** ^1^ Department of Immunology, Faculty of Medicine and Dentistry, Palacky University Olomouc, Olomouc, Czech Republic; ^2^ Department of Hemato-Oncology, Faculty of Medicine and Dentistry, Palacky University and University Hospital, Olomouc, Czech Republic; ^3^ Department of Computer Science, Faculty of Electrical Engineering and Computer Science, VSB-Technical University of Ostrava, Ostrava, Czech Republic

**Keywords:** serum pattern, cytokines, growth factors, proximity extension immunoassay, post-transplant serum pattern

## Abstract

Serum protein fingerprints associated with MGUS and MM and their changes in MM after autologous stem cell transplantation (MM-ASCT, day 100) remain unexplored. Using highly-sensitive Proximity Extension ImmunoAssay on 92 cancer biomarkers (Proseek Multiplex, Olink), enhanced serum levels of Adrenomedullin (ADM, *P*_corr_= .0004), Growth differentiation factor 15 (GDF15, *P*_corr_= .003), and soluble Major histocompatibility complex class I-related chain A (sMICA, *P*_corr_= .023), all prosurvival and chemoprotective factors for myeloma cells, were detected in MM comparing to MGUS. Comparison of MGUS and healthy subjects revealed elevation of angiogenic and antia-poptotic midkine (*P*_corr_= .0007) and downregulation of Transforming growth factor beta 1 (TGFB1, *P*_corr_= .005) in MGUS. Importantly, altered serum pattern was associated with MM-ASCT compared to paired MM at the diagnosis as well as to healthy controls, namely by upregulated B-Cell Activating Factor (sBAFF) (*P*_corr_< .006) and sustained elevation of other pro-tumorigenic factors. In conclusion, the serum fingerprints of MM and MM-ASCT were characteristic by elevated levels of prosurvival and chemoprotective factors for myeloma cells.

## INTRODUCTION

Monoclonal gammopathy of undetermined significance (MGUS) is a precursor lesion to overt multiple myeloma (MM), a clonal B-cell malignancy characterized by excessive multiplication of a plasma cell clone(s) in bone marrow, and accumulation of either a monoclonal immunoglobulin (Ig) (M-protein) or an Ig-free light chain in blood [1]. During the last decade, MM treatment and patient outcomes improved remarkably after the introduction of novel agents and autologous stem cell transplantation (ASCT) [2]. However, even with the best combination of currently available drugs, a cure is not achieved for most MM patients [2, 3]. Better characterization of neoplastic cells and microenvironment in particular myeloma stages is therefore needed as well as clarifying of reason(s) for treatment failure in most MM patients [2, 3, 4]. The neoplastic plasma cells in MGUS and MM share similar genetic abnormalities, probably occurring as early events [5, 6]. The key role in microenvironment play bone marrow stromal cells and other microenvironmental cells that secrete a plethora of cytokines and growth factors after paracrine stimulation and/or direct interaction with neoplastic cells [7]. Moreover, also myeloma cells secrete numerous cytokines and growth factors [8, 9]. The secreted molecules may, in turn, promote homing, migration, proliferation, survival of malignant plasma cells as well as contribute to the bone resorption and drug resistance [10].

Given the key role of cytokines and growth factors in MM pathogenesis, we investigated the complexity of serum microenvironment using novel multiplex highly-sensitive PEA immunoassay on 92 cancer-related proteins followed by pattern-recognition analyses. Besides identification of serum fingerprints distinguishing MGUS and MM, we for the first time compared paired MM samples from the time of diagnosis and after autologous stem cell transplantation (MM-ASCT) as well as MGUS to healthy subjects.

## RESULTS

### Serum protein fingerprinting in MGUS and MM by PEA immunoassay

**Figure 1:**
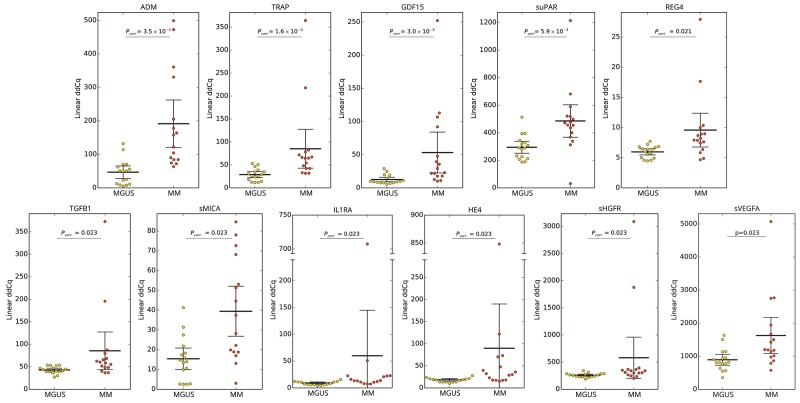
Distribution of serum levels of top-ranked proteins in patients with MGUS *vs* MM Group means are indicated by horizontal bars, error bars indicate 95%CI; *P*_*corr*_ values for differences between two groups of patients after multiple corrections are stated.

**Figure 2:**
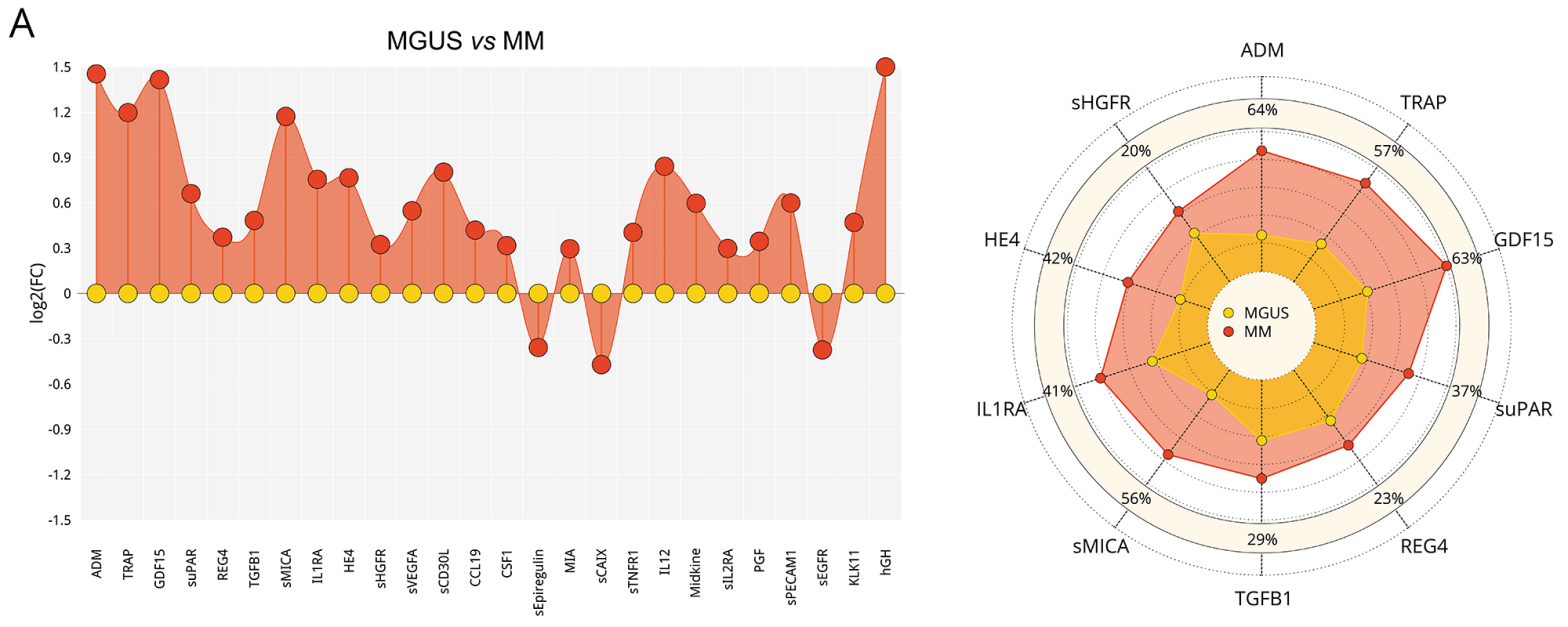

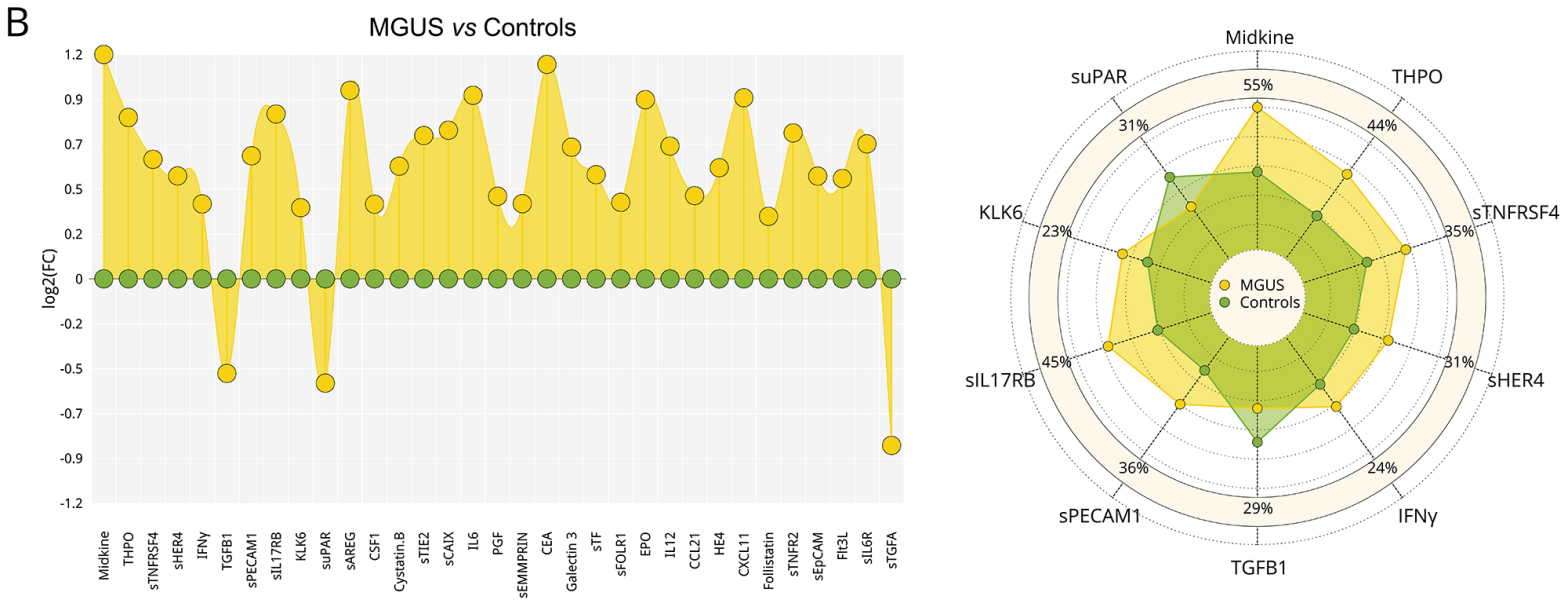

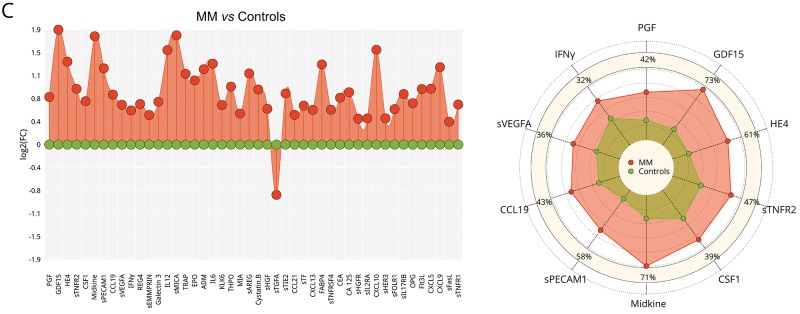

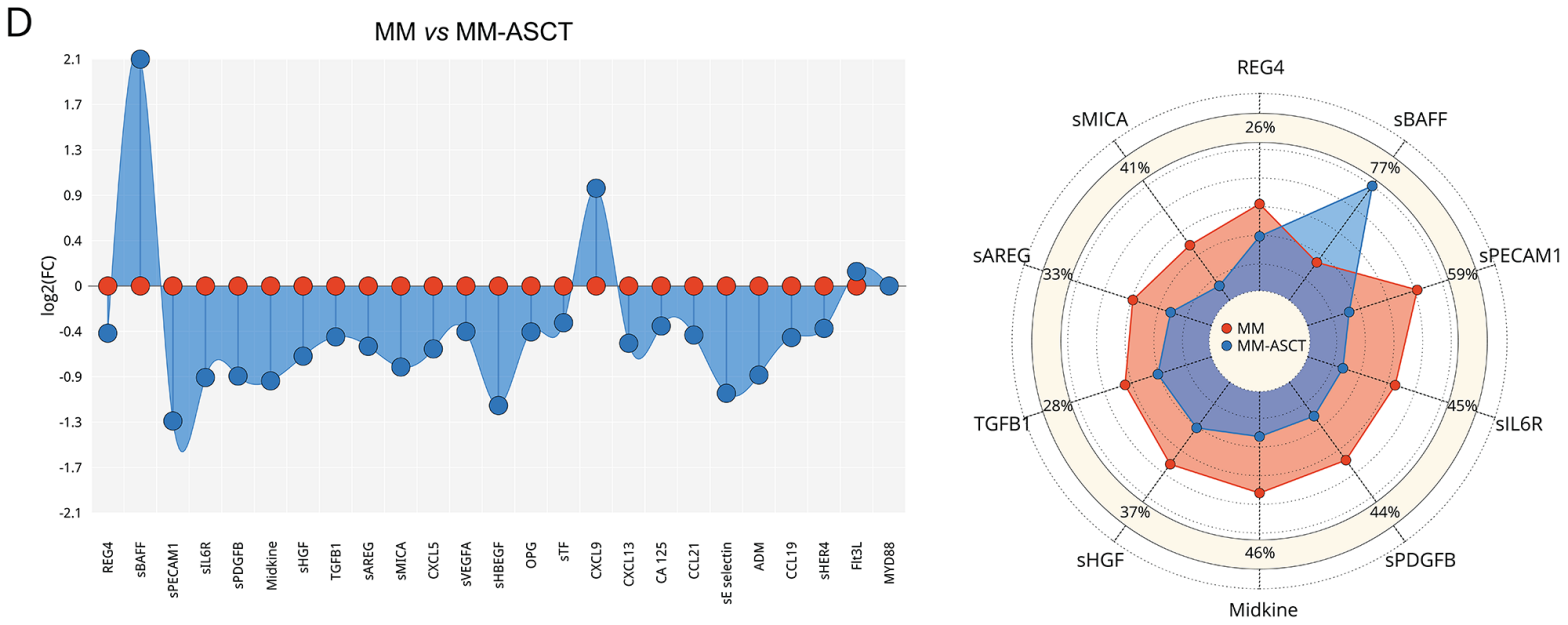

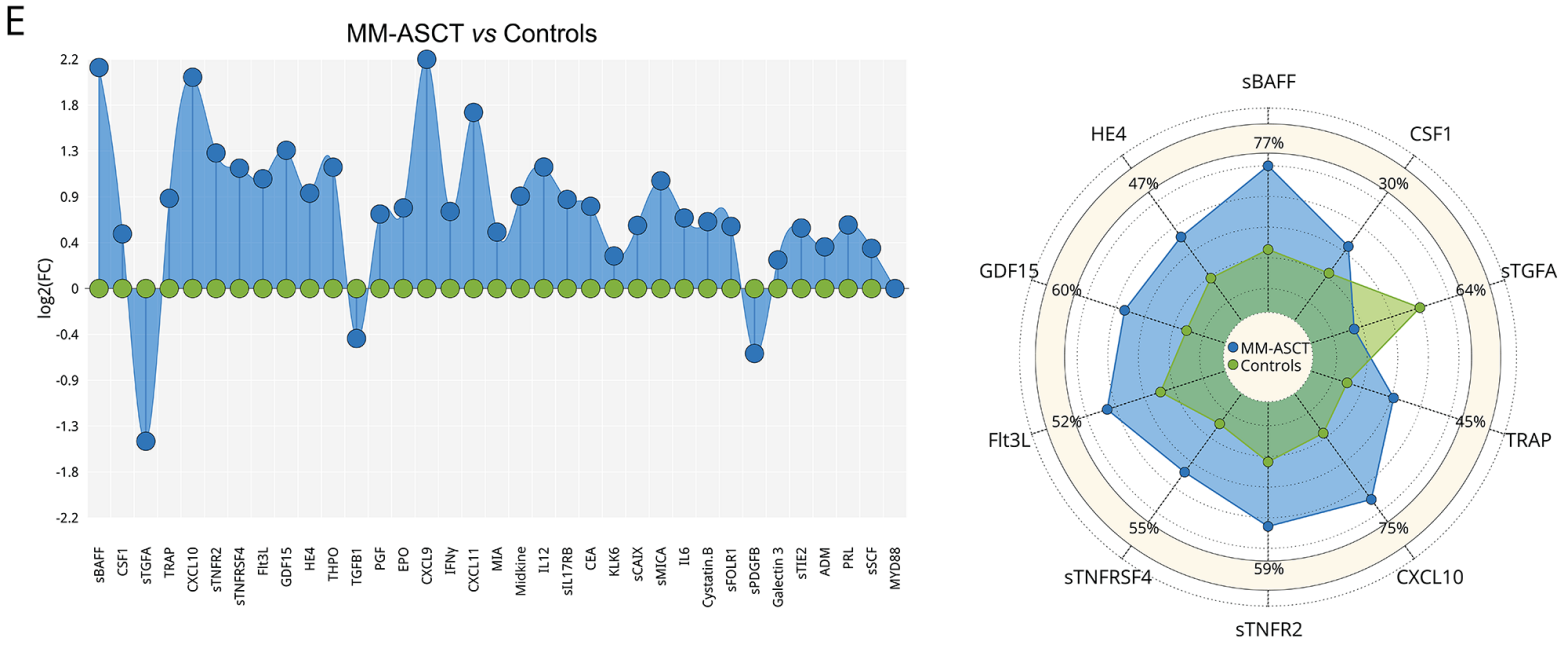
Serum fingerprints and changes in top-deregulated proteins in MGUS, MM, and MM-ASCT Fingerprints were presented as FC (fold-change of group medians) of serum levels of deregulated serum proteins between particular groups (*P*< .05); changes in top-deregulated proteins are presented as percentage of changes between group medians of particular groups: **A**. MGUS *vs* MM, **B**. controls *vs* MGUS, **C**. controls *vs* MM. **D**. MM *vs* MM-ASCT and **E.** controls *vs* MM-ASCT. MGUS is colored yellow, MM red, MM-ASCT blue, and control subjects green.

To assess the serum protein fingerprints associated with MGUS and MM, we compared serum protein pattern obtained by PEA immunoassay in MGUS and MM and healthy controls. Of ninety-two analyzed biomarkers (Supplementary Table S1), levels of six biomarkers (sBTC, CA242, sER, GM-CSF, IL2, IL4) were below the Proseek limit of detection (LOD) in all studied groups and were therefore excluded from further analysis. Comparing MGUS and MM, 26 analytes were deregulated between these groups, whereas 13 analytes reached the significance after the adjustment for multiple comparisons (Supplementary Table S2A). The distribution of serum levels of top-ranked proteins (ADM, TRAP, GDF15, suPAR, REG4, TGFB1, sMICA, IL1RA, HE4, sHGFR, sVEGFA; see Table 1A), all found upregulated in MM, is shown in Figure 1. The protein serum fingerprints associated with MGUS and MM and the changes in protein levels between MGUS and MM for top-deregulated analytes are shown in Figure 2A.

**Table 1:** Serum levels of top-ranked proteins differentiating between A) MGUS *vs* MM, B) healthy controls *vs* MGUS, C) healthy controls *vs* MM, D) MM *vs* MM-ASCT and E) healthy controls *vs* MM-ASCT.

Analyte	Mean Linear ddCq (95% CI)		FC	*P*	*P*_*corr*_
**A**	*MGUS*	*MM*			
ADM	46.9 (28.4-65.4)	191 (121-262)	2.80	4.0 × 10^-6^	3.5 × 10^-4^
TRAP	28.9 (22.3-35.6)	85.1 (42.8-127)	2.33	3.7 × 10^-5^	1.6 × 10^-3^
GDF15	12.6 (9.19-16.0)	53.3 (22.5-84.1)	2.72	1.1 × 10^-4^	3.0 × 10^-3^
suPAR	295 (251-338)	486 (368-603)	1.60	2.7 × 10^-4^	5.9 × 10^-3^
REG4	5.96 (5.46-6.46)	9.58 (6.78-12.4)	1.30	1.2 × 10^-3^	.021
TGFB1	42.9 (39.0-46.8)	85.5 (43.7-127)	1.41	1.9 × 10^-3^	.023
sMICA	15.4 (9.98-20.8)	39.4 (26.8-52.1)	2.29	2.1 × 10^-3^	.023
IL1RA	9.22 (7.23-11.2)	60.0 (0-145)	1.71	2.6 × 10^-3^	.023
HE4	18.4 (15.6-21.3)	89.8 (0-190)	1.72	3.0 × 10^-3^	.023
sHGFR	259 (240-278)	580 (199-960)	1.26	3.0 × 10^-3^	.023
sVEGFA	892 (728-1056)	1628 (1082-2173)	1.47	3.0 × 10^-3^	.023
**B**	*healthy controls*	*MGUS*			
Midkine	30.3 (25.8-34.8)	75.0 (60.8-89.3)	2.24	8.2 × 10^-6^	7.0 × 10^-4^
THPO	13.1 (11.7-14.6)	24.2 (18.8-29.6)	1.79	9.8 × 10^-5^	4.2 × 10^-3^
sTNFRSF4	4.12 (3.29-4.94)	7.39 (6.05-8.72)	1.54	1.6 × 10^-4^	4.4 × 10^-3^
sHER4	174 (163-184)	270 (228-311)	1.45	2.4 × 10^-4^	4.5 × 10^-3^
IFNγ	1.58 (1.52-1.64)	2.17 (1.95-2.39)	1.31	3.1 × 10^-4^	4.5 × 10^-3^
TGFB1	61.9 (54.2-69.5)	42.9 (39.0-46.8)	.71	3.7 × 10^-4^	4.5 × 10^-3^
sPECAM1	15.6 (12.6-18.7)	28.7 (23.3-34.1)	1.56	3.7 × 10^-4^	4.5 × 10^-3^
sIL17RB	5.56 (3.84-7.28)	10.9 (9.09-12.7)	1.81	1.1 × 10^-3^	.010
KLK6	28.6 (24.5-32.8)	44.0 (37.9-50.2)	1.29	1.1 × 10^-3^	.010
suPAR	425 (373-476)	295 (251-338)	.69	2.0 × 10^-3^	.017
**C**	*healthy controls*	*MM*			
PGF	59.2 (52.5-66.0)	137 (97.2-177)	1.72	1.6 × 10^-5^	1.4 × 10^-3^
GDF15	8.34 (6.14-10.5)	53.3 (22.5-84.1)	3.69	9.8 × 10^-5^	4.2 × 10^-3^
HE4	12.9 (10.4-15.5)	89.8 (0-190)	2.56	1.6 × 10^-4^	4.4 × 10^-3^
sTNFR2	9.52 (7.93-11.1)	17.9 (14.3-21.6)	1.88	2.4 × 10^-4^	5.3 × 10^-3^
CSF1	100 (94.4-106)	195 (115-275)	1.64	3.7 × 10^-4^	5.3 × 10^-3^
Midkine	30.3 (25.8-34.8)	158 (82.9-232)	3.42	3.7 × 10^-4^	5.3 × 10^-3^
sPECAM1	15.6 (12.6-18.7)	69.6 (17.4-122)	2.38	7.7 × 10^-4^	9.3 × 10^-3^
CCL19	463 (317-609)	929 (733-1125)	1.76	1.1 × 10^-3^	9.3 × 10^-3^
sVEGFA	765 (700-830)	1628 (1082-2173)	1.57	1.1 × 10^-3^	9.3 × 10^-3^
IFNγ	1.58 (1.52-1.64)	5.17 (0-11.1)	1.47	1.1 × 10^-3^	9.3 × 10^-3^
**D**	*MM*	*MM-ASCT*			
REG4	9.58 (6.78-12.4)	7.01 (4.50-9.51)	.74	3.1 × 10^-5^	2.6 × 10^-3^
sBAFF	18.4 (10.5-26.3)	62.3 (51.8-72.9)	4.38	1.5 × 10^-4^	6.1 × 10^-3^
sPECAM1	69.6 (17.4-122)	17.3 (13.1-21.6)	.41	2.1 × 10^-4^	6.1 × 10^-3^
sIL6R	174 (99.3-249)	81.6 (64.6-98.6)	.55	5.8 × 10^-4^	.012
sPDGFB	544 (299-789)	274 (222-327)	.56	7.6 × 10^-4^	.013
Midkine	158 (82.9-232)	54.5 (42.7-66.3)	.54	1.0 × 10^-3^	.014
sHGF	47.1 (32.5-61.6)	26.9 (20.7-33.1)	.63	1.3 × 10^-3^	.014
TGFB1	85.5 (43.7-127)	41.6 (34.9-48.3)	.72	1.3 × 10^-3^	.014
sAREG	19.0 (5.32-32.8)	7.52 (6.17-8.87)	.67	1.7 × 10^-3^	.016
sMICA	39.4 (26.8-52.1)	22.5 (15.8-29.2)	.59	2.1 × 10^-3^	.018
**E**	*healthy controls*	*MM-ASCT*			
sBAFF	14.1 (12.9-15.3)	62.3 (51.8-72.9)	4.32	8.2 × 10^-6^	7.0 × 10^-4^
CSF1	100 (94.4-106)	152 (131-173)	1.44	2.4 × 10^-4^	6.0 × 10^-3^
sTGFA	24.4 (19.6-29.2)	11.9 (9.03-14.8)	.36	2.4 × 10^-4^	6.0 × 10^-3^
TRAP	32.2 (25.0-39.4)	71.2 (42.5-100.0)	1.82	3.7 × 10^-4^	6.0 × 10^-3^
CXCL10	130 (61.2-199)	667 (323-1011)	4.05	5.4 × 10^-4^	6.0 × 10^-3^
sTNFR2	9.52 (7.93-11.1)	21.2 (17.6-24.9)	2.45	5.4 × 10^-4^	6.0 × 10^-3^
sTNFRSF4	4.12 (3.29-4.94)	10.9 (7.21-14.6)	2.22	5.4 × 10^-4^	6.0 × 10^-3^
Flt3L	254 (225-284)	522 (411-633)	2.07	7.7 × 10^-4^	6.0 × 10^-3^
GDF15	8.34 (6.14-10.5)	22.7 (16.5-28.8)	2.50	7.7 × 10^-4^	6.0 × 10^-3^
HE4	12.9 (10.4-15.5)	43.6 (3.61-83.5)	1.88	7.7 × 10^-4^	6.0 × 10^-3^
THPO	13.1 (11.7-14.6)	28.5 (21.7-35.3)	2.23	7.7 × 10^-4^	6.0 × 10^-3^

FC (Fold Change) between group medians of linear ddCq

*P_*corr*_ value corrected for multiple comparisons (Benjamini-Hochberg correction)

Comparison of protein pattern obtained in MGUS and healthy controls revealed deregulation of 33 proteins (Figure 2B), of these 21 reached significance after multiple comparisons (Supplementary Table S2B). The protein levels of top-ranked proteins (midkine, THPO, sTNFRSF4, sHER4, INFγ, TGFB1, sPECAM1, sIL17RB, KLK6, suPAR) are presented in Table 1B and Supplementary Figure S1A.

When comparing MM and controls, we observed deregulation of 46 serum proteins (Figure 2C), of these 41 analytes reached significance after adjustment for multiple comparisons (Supplementary Table S2C). The distribution of serum levels of top-ranked proteins between MM and controls (PGF, GDF15, HE4, sTNFR2, CSF1, midkine, sPECAM1, CCL19, sVEGFA, INFγ; see Table 1C) is shown in Supplementary Figure S1B. The subanalysis based on cytogenetic/FISH analysis was not performed due to the high heterogeneity within the group.

### Changes in serum protein pattern in post-transplant MM

**Figure 3:**
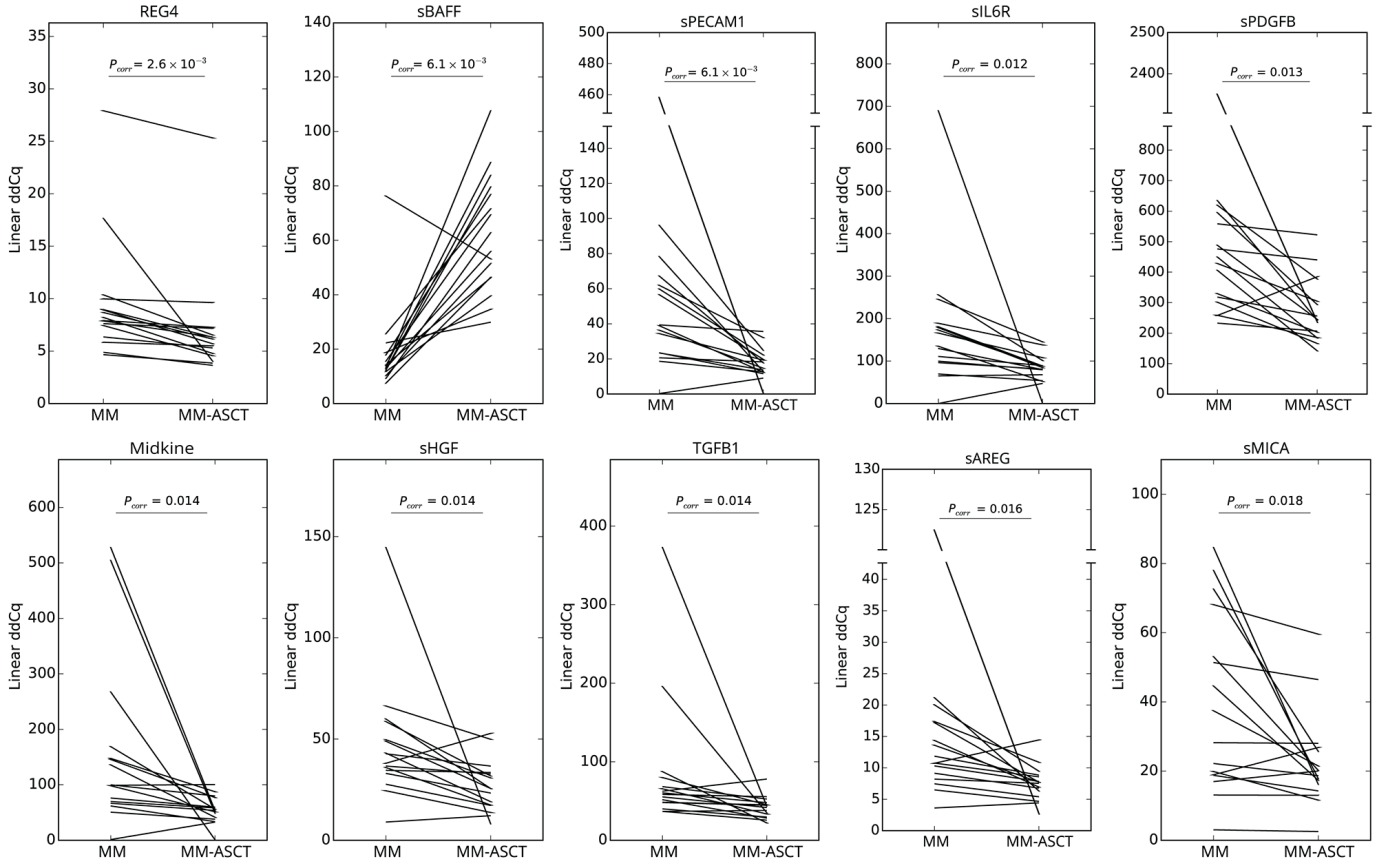
Changes in serum protein levels between paired samples from MM at the diagnosis and after ASCT (day 100) *P*_*corr*_ values for differences between two groups of patients after multiple corrections are stated.

To assess the changes in serum protein pattern in MM after ASCT, we compared the post-transplant sera (day 100) with paired samples obtained in MM patients at the time of diagnosis and healthy control subjects. Comparing paired samples from MM-ASCT and MM, the most upregulated protein in post-transplant sera was sBAFF (*P*_*corr*_= .006), followed by CXCL9 (*P*_*corr*_= .041). Next twenty-one proteins were downregulated (14 proteins after adjustment for multiple comparisons) in MM-ASCT comparing to MM (Figure 2D, Supplementary Table S2D). The top-ranked proteins were: elevated sBAFF and downregulated REG4, sPECAM1, sIL6R, sPDGFB, midkine, sHGF, TGFB1, sAREG, and sMICA in MM-ASCT comparing to MM (Table 1D, Figure 3). Importantly, serum levels of MM-associated pro-tumorigenic factors such as GDF15, CSF1, suPAR, and HE4 did not change after ASCT comparing to sample at the diagnosis (Supplementary Table S2D).

To exclude the influence of treatment regime on serum pattern, we assessed the protein profile in subgroups based on ASCT induction regime (IMiD-based/bortezomib-based). We did not detect any differences in the cytokine levels as a function of the induction regime as well as the hematological response (CR, VGPR/PR) on day 100 (*P*_*corr*_> .05).

**Figure 4:**
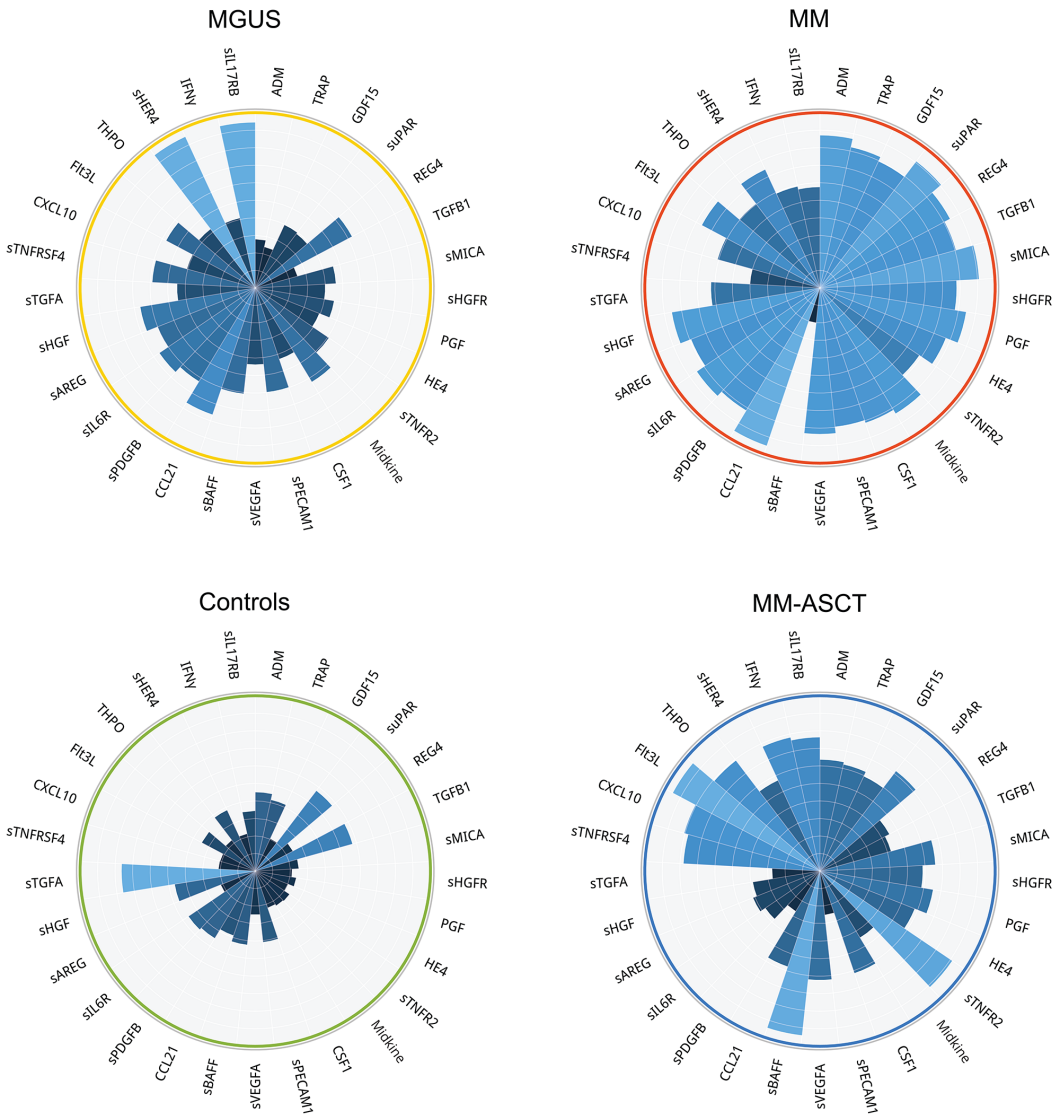
Comparison of protein fingerprints in MGUS, MM, MM-ASCT and healthy subjects for selection of top-deregulated proteins

Comparing to healthy subjects, the serum of post-transplant MM patients showed permanently altered pro-tumorigenic signature characteristic by deregulation of 35 proteins (after multiple adjustments: 28 analytes) (Figure 2E, Supplementary Table S2E). Except sTGFA and TGFB1, all deregulated proteins were elevated in MM-ASCT. The top-ranked proteins between MM-ASCT and healthy controls were as follows: sBAFF, CSF1, sTGFA, TRAP, CXCL10, sTNFR2, sTNFRSF4, Flt3L, GDF15, HE4, THPO (Table 1E, Supplementary Figure S1C). The serum protein pattern in MM-ASCT and its comparison to those of healthy controls, MGUS and MM, are presented in Figure 4.

### Pattern-recognition algorithms

**Figure 5:**
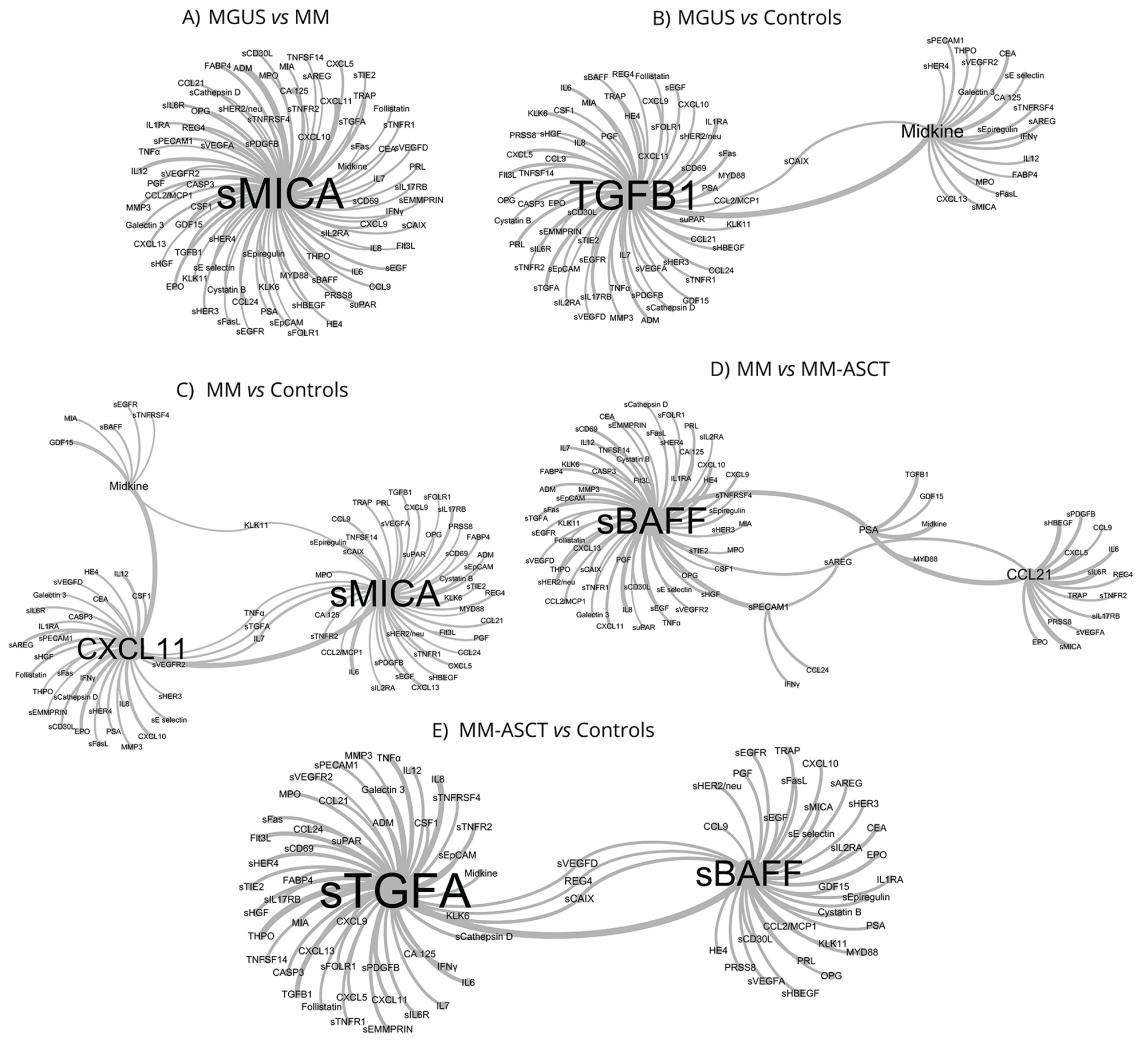
Network visualization of classification models obtained by pattern-recognition analysis that identified key serum biomarkers distinguishing between MGUS, MM, and MM-ASCT based on co-occurrence of analytes in classification models **A.** MGUS *vs* MM, **B.** controls *vs* MGUS, **C.** controls *vs* MM, **D.** MM *vs* MM-ASCT and **E.** controls *vs* MM-ASCT. The size of the vertices (font-size) and connections among vertices show those proteins, which were used in classification rules of the particular patient group in the most accurate classification model.

To facilitate the selection of the most promising circulating proteins distinguishing studied groups (MGUS, MM, MM-ASCT, healthy subjects), we applied advanced binary classification algorithm and analyzed co-occurrence of analytes in classification models. The most accurate classification model for separation of MGUS and MM utilized in classification rules most frequently sMICA in combination with other analytes (Figure 5A). In MGUS *vs* healthy controls, the classification rules used most often TGFB1 and midkine (Figure 5B) and in MM *vs* healthy controls most often sMICA, CXCL11, and midkine (Figure 5C). The classification model for MM and MM-ASCT used in the classification rules most frequently sBAFF and CCL21 (Figure 5D) and for MM-ASCT and controls used sTGFA and sBAFF (Figure 5E).

### Classification of MGUS, MM, and MM-ASCT

**Figure 6:**
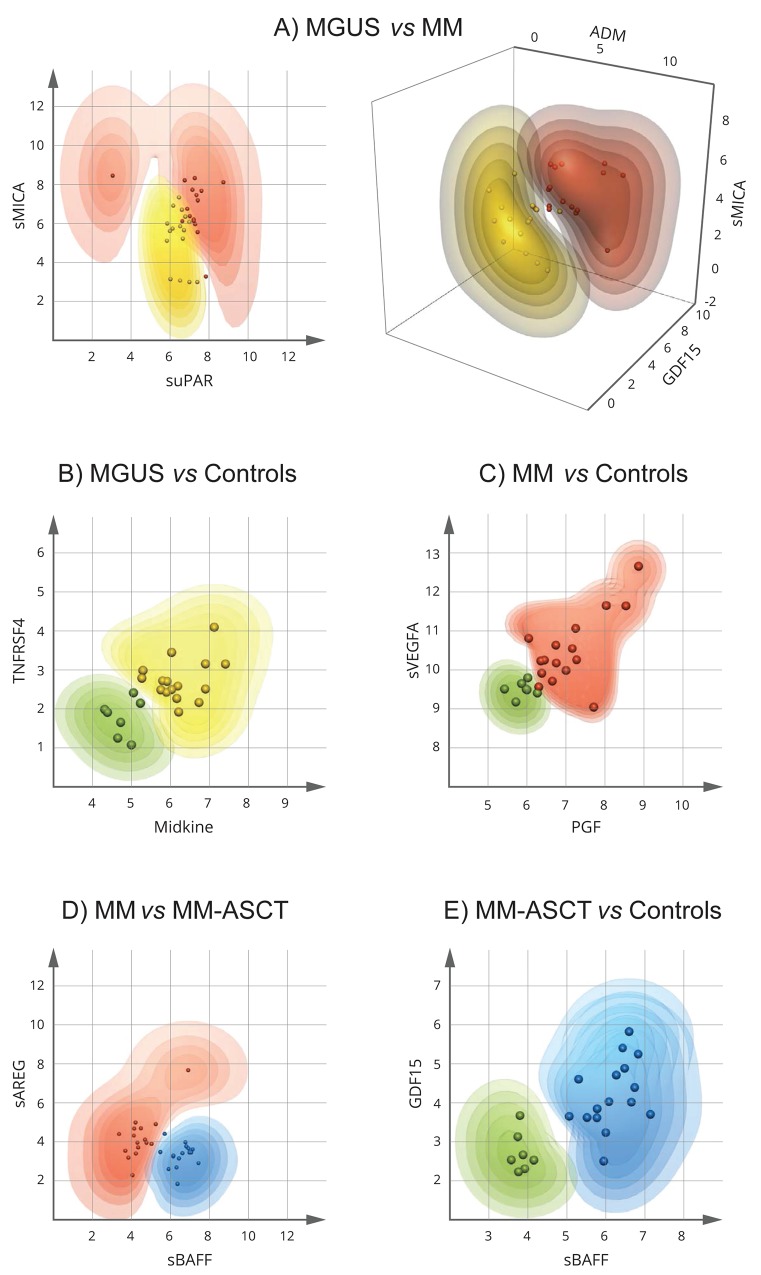
Extended Support Vector Machine (kSVM) analysis for identification of the minimum number and the best combination of proteins distinguishing MGUS, MM, and MM-ASCT The dots represent the individual patient data (combinations of two or three analytes). The contour plots show the probabilities (intervals: > 90, 90-80, 80-70, 70-60, and 60-50%) for input data from individual patients to belong to the particular patient group. MGUS is colored yellow, MM red, MM-ASCT blue, and control subjects green. The more saturated color the higher probability of correct classification. **A.** MGUS *vs* MM, **B.** controls *vs* MGUS, **C.** controls *vs* MM **D.** MM *vs* MM-ASCT, **E.** controls *vs* MM-ASCT.

To detect the minimum number and the best combination of serum analytes able to discriminate between MGUS and MM, and MM-ASCT, we applied Multilinear Discriminant Analysis, Naive Bayes classifiers, Random Forests, and extended Support Vector Machine (kSVM). The probability of correct classification to particular patient subgroup (intervals: >90, 90-80, 80-70, 70-60, and 60-50%) was calculated for every combination of two or three analytes from individual patients, and the misclassification error was determined. The best visual separation of studied patient groups was achieved by kSVM and therefore used in further study. The best dual-combination able to discriminate MGUS *vs* MM was achieved by the combination of sMICA and suPAR, able to separate these groups with a classification error of 0.062 (1 false/16 samples) (Figure 6A). The best triple-combinations for separating MGUS and MM were sMICA-ADM-GDF15 (Figure 6A) as well as the combination of sMICA-ADM-REG4, sMICA-suPAR-REG4, sMICA-suPAR-sHGFR, ADM-suPAR-REG4, TRAP-REG4-sHGFR (data not shown). The triple-combinations increased the probability of correct classification of MGUS and MM; the classification error remained 0.062 (1 false/16). For discrimination of MGUS and MM, MM-ASCT from controls and MM from MM-ASCT, several combinations of only two analytes were sufficient to classify all samples correctly (with no misclassification error). For MGUS *vs* controls, the combinations were as follows: midkine-sTNFRSF4 (Figure 6B) or midkine-TGFB1, TGFB1-THPO, TGFB1-sHER4, TGFB1-IFNγ, and TGFB1-sIL17RB (data not shown). Best separation of MM and controls was observed for combinations PGF-sVEGFA (Figure 6C) and PGF-midkine. Regarding serum from MM and MM-ASCT, the best analyte pairs able to discriminate these groups were sBAFF-sAREG (Figure 6D) or the combination of sBAFF with sPECAM1 or sHGF (data not shown). For MM-ASCT *vs* controls, the combination of following analyte pairs resulted in 100% correct separation: sBAFF-GDF15 (Figure 6E) or sBAFF in combination with other analytes such as CSF1, sTGFA, TRAP, sTNFR2, sTNFRSF4 or HE4. Also, the combination of sTGFA with CSF1, sTNFR2 or Flt3L discriminated fully between MM-ASCT and healthy controls (data not shown).

## DISCUSSION

In this study, we investigated the complexity of serum microenvironment in MGUS, MM and MM after ASCT using highly-sensitive PEA immunoassay. We hypothesized that serum of pre-cancer MGUS and MM differ by the presence of pro-tumorigenic factors. Indeed, we detected elevated levels of adrenomedullin (ADM), TRAP, GDF15, TGFB1, suPAR and other pro-tumorigenic proteins in serum of MM patients compared to MGUS. These proteins were already reported in MM but not investigated simultaneously. Pro-angiogenic factor ADM was identified as the most highly upregulated gene in hypoxia-dependent/independent fashion in MM cells, suggesting to be a major driving force for the angiogenic switch during MM evolution [11]. Highly upregulated TRAP is a marker of osteoclasts driving the bone resorption in MM [12]. The crucial role of TGFB1 in MM is supported by the observation that the inhibition of TGF-β signaling by TGF-β type I receptor kinase inhibitor causes a suppression of MM cell growth and an enhancement of bone formation [13]. Regarding GDF15 in MM, high serum levels were associated with poor prognosis [14, 15] and treatment response [16] and osteolysis [17]. GDF15 enhances the tumor-initiating and self-renewal potential of myeloma cells [18], contributes to drug resistance in both stroma-dependent/independent MM cells [14, 15], and promotes osteoclast differentiation while inhibits osteoblast differentiation [17]. Regarding suPAR, high suPAR expression in MM predicts progression, shorter survival and early extramedullary infiltration [19].

In order to detect the most promising circulating protein(s) distinguishing MGUS and MM, we analyzed co-occurrence of analytes in MGUS/MM classification models and identified sMICA as the most useful classifier. High serum levels of sMICA were already detected in MM as an adverse prognostic factor [20, 21], but not elevated in MGUS [20]. sMICA may originate from MICA-expressing MM cells, fibroblasts or other stromal cells upon stimulation [8, 22]. There is evidence that sMICA impairs the function of the NKG2D + T CD8+ and NK cells, contributing to myeloma cell immune escape [20]. Additionally, patients with MGUS, but not MM, generate high-titer anti-MICA antibodies that antagonize the suppressive effects of sMICA [20]. It has been therefore suggested that alterations in the NKG2D pathway by sMICA and anti-MICA antibodies are critically involved in the suppression of innate and adaptive immunity during the progression from MGUS to MM [20]. Importantly, some drugs may reconstitute the capabilities of sMICA-inhibited cytotoxicity of CD8+ and NK cells [23, 24], thus further highlights the potential of NKG2D + T CD8+ and NK cell-mediated immunotherapeutic interventions in MM [24, 25, 26].

Next, we investigated the minimum number and best combination of serum analytes able to discriminate between MGUS and MM. Advanced data mining methods revealed that the combination of sMICA and suPAR separates these groups with a classification error 0.062. The combination of triplets sMICA-ADM-GDF15 or sMICA-suPAR-REG4 increased the probability of correct classification of MGUS and MM with the same classification error (1/16) significantly. Although larger cohort studies are needed to confirm our results, our study nominated sMICA, ADM, GDF15, suPAR, and REG4 as key MM-associated serum proteins able to discriminate MGUS and MM.

Despite new therapies and ASCT increasing remission rates, nearly all MM patients ultimately succumb to disease relapse and progression. Because tumor microenvironment may contribute to these processes, we investigated for the first time serum pattern from paired samples from MM patients from the time of diagnosis and after ASCT (day 100). Interestingly, the post-transplant sera possessed high levels of soluble B-Cell Activating Factor (sBAFF), a survival factor for myeloma cells [9]. Enhanced serum levels of sBAFF, found produced by MM cells, immune and stromal cells [9, 27], correlated inversely with overall survival in MM and resistance to dexamethasone and lenalidomide [27, 28]. Since the elimination of sBAFF in an MM mouse model resulted in a decrease of tumor burden and protected against lytic bone disease [29], the sBAFF signaling represents a promising therapeutic target in MM [27], especially in the setting of post-transplant sBAFF elevation. After ASCT, the MM-associated proteins sMICA and ADM were downregulated but still elevated compared to healthy controls. On the other hand, serum levels of other pro-tumorigenic factors such as GDF15, CSF1, suPAR, and others did not change after ASCT comparing to paired MM sample at the diagnosis. Similar observation was reported in treated MM patients showing that cytokine pattern in those achieving remission is not restored to physiological levels [30], thus suggesting that once an individual has MM, the microenvironment is permanently altered and primed for a relapse. These results highlight the role of microenvironment for treatment success and may explain why MM remains an incurable disease.

We were also interested in MGUS associated serum pattern comparing to healthy controls. Our analysis revealed for the first time that MGUS is characteristic by low levels of TGFB1 and high levels of midkine, a heparin-binding growth factor involved in angiogenic and anti-apoptotic functions and tumor expansion in various cancers [31, 32]. Enhanced gene expression of midkine and other angiogenic factors were already reported in MM [33, 34] and also in this study we detected higher serum levels of midkine in MM *vs* MGUS. Importantly, lower gene expression of midkine and other angiogenic genes was detected in IMiD-responders compared to non-responders [34]. Elevation of midkine, produced by normal and malignant B-cells, tumor and stromal cells [35, 36], was also reported in other B-cell malignancies such as chronic lymphocytic leukemia and lymphomas [35]. Regarding TGFB1, low levels of TGFB1 were shown to control MM cell growth [13]. Our observations highlight the role of TGFB1 and midkine in the progression of MGUS to MM thus deserving further investigation.

We are aware that this study has several limitations. Because this study was focused on determination of serum protein fingerprinting, we did not analyze plasma bone marrow and did not investigate the functional effect of deregulated proteins. This should be performed in future studies.

In conclusion, we identified serum protein fingerprints associated with MGUS and MM as well changes ongoing in MM after ASCT. The knowledge of serum pattern may contribute to the identification of key myeloma cell survival factors, which may in turn influence treatment response and disease development.

## MATERIALS AND METHODS

### Study population and materials

The study cohort includes patients with MGUS (n=16) and MM (n=16); all patients were diagnosed according to the criteria of International Myeloma Working Group [37, 38]. Serum samples were taken at the time of diagnosis from previously untreated patients, aliquoted and stored at -80°C until analysis. In all enrolled MM patients, paired serum sample collected at day 100 after ASCT (MM-ASCT) was also analyzed. Patient characteristics are described in Table 2. Serum samples from healthy control subjects (n=7, mean age 51 yrs; range 45-72 yrs, 4 males/3 females) were taken from members of medical staff; all completed a questionnaire regarding their health status. All patients provided written informed consent about the usage of peripheral blood for the purpose of this study. The study was approved by the ethics committee of University Hospital and Palacky University Olomouc.

**Table 2:** Patient characteristics.

Parameter	MGUS (n=16)	MM (n=16)
Age, years, median (min-max)	59 (46-83)	57 (39-64)
Gender, n, male/female	8/8	5/11
Paraprotein type, n (%)		
IgG	11 (69)	7 (44)
IgA	4 (25)	4 (25)
IgD	1 (6)	0 (0)
Light chain only	0 (0)	5 (31)
Bone lesions, n, yes/no	0/16	12/4
ASCT Induction regime, n (IMiD-based/bortezomib-based)*	NA	16 (8/8)
Time difference from diagnosis to day 100 after ASCT, days, mean (min-max)	NA	314 (231-567)
Response on day 100 after ASCT, CR/VGPR/PR, n (%)	NA	11/4/1 (69/25/6)

*1 patient received tandem ASCT

NA not applicable, CR complete remission, VGPR very good partial remission, PR partial remission

### Proximity extension immunoassay

Serum profiles of ninety-two cancer-related proteins were assessed by the Proseek Multiplex Oncology I kit (Olink Bioscience, Uppsala, Sweden) according to the manufacturer’s recommendation. Briefly, serum samples (1 µl) were incubated in the presence of 92 proximity antibody pairs tagged with DNA reporter molecules. Once the pair of antibodies bound to their corresponding antigens, the respective DNA tails formed by proximity extension an amplicon that was quantified by high-throughput real-time PCR (BioMark™ HD System, Fluidigm Corporation). The generated fluorescent signal directly correlates with protein abundance. Olink Wizard (Olink) was used for data normalization: the raw Cq-value (log2 scale) for each data point was normalized by subtracting the Cq-value for the extension control and compared to that of the corresponding background reaction resulting in a ddCq-value [39]. For further analysis, linearized values (2^ddCq^) were used. For panel description see Supplementary Table S1, for sensitivity and specificity parameters of PEA analysis see Assarsson et al. [39].

### Pattern-recognition algorithms

Binary classification by a stochastic nature-inspired symbolic regression method and evolutionary fuzzy-rules [40] was conducted in order to learn symbolic models for particular patient groups (MGUS, MM, MM-ASCT, healthy subjects) based on their serum protein pattern. For each tested patient group pair, the procedure was repeated more than 500 times to accommodate the stochastic nature of the algorithm and to obtain representative results. The most accurate classification models, separating all patients in the correct patient groups, were utilized for the identification of key molecules and those co-occurring in the classification rules characteristic for the particular patient group.

The proteins from the classification rules were further used to form a network model of patient classification with molecules as vertices and co-occurrences in successful classification models as edges. An algorithm based on analysis of the nearest neighbors between the studied molecules was applied to determine vertex and edge weights in the network model [41]. The size of the vertices (font-size) and connections among vertices show those proteins, which were used in classification rules of the particular patient group in the most accurate classification model.

### Classification methods for separation of patient groups

To assess the minimum number and best combination of serum analytes distinguishing between the patient groups (MGUS *vs* MM, MM *vs* MM-ASCT), we applied several classification methods from the area of information retrieval such as Multilinear Discriminant Analysis, Naive Bayes classifiers, Random Forests, and kSVM to calculate the class probabilities for every input data (combinations of two or three analytes) and to determine the misclassification error [42, 43]. All methods were calculated using R statistical software with package Caret (http://topepo.github.io/caret/index.html).

### Statistical analysis

Statistical analyses (Mann-Whitney-Wilcoxon and paired Wilcoxon tests) were performed using R statistical software package (http://www.r-project.org/). All data analyses were performed on linearized expression data (2^ddCq^) for each protein. A combination of box plots and swarm plots (a one-dimensional hybrid between scatter plot and strip chart) was employed to visualize the distribution of signals across subjects in particular subgroups. Radar charts were created for each pair of compared subgroups to visually assess the quantitative changes in levels of the most significant molecules, determined for each group pair by the Mann-Whitney-Wilcoxon test, and for paired samples (MM and MM-ASCT) by paired Wilcoxon test. *P*-value for each protein was adjusted for multiple comparisons using the False Discovery Rate (FDR) by the Benjamini-Hochberg procedure. A *P*-value < .05 was considered as significant.

## SUPPLEMENTARY MATERIALS FIGURES AND TABLES







## Abbreviations

ADM: Adrenomedullin
ASCT: Autologous stem cell transplantation
CA125: Cancer antigen 125
CA242: CA 242 tumor marker
CCL19: Chemokine (C-C motif) ligand 19
CCL21: Chemokine (C-C motif) ligand 21
CEA: Carcinoembryonic antigen
CR: Complete remission
CSF1: Colony stimulating factor 1
CXCL5: C-X-C motif chemokine 5
CXCL9: Chemokine (C-X-C motif) ligand 9
CXCL10: C-X-C motif chemokine 10
CXCL11: C-X-C motif chemokine 11
CXCL13: Chemokine (C-X-C motif) ligand 13
EPO: Erythopoietin
FABP4: Fatty acid binding protein 4
FDR: False Discovery Rate
Flt3L: FMS-like tyrosine kinase 3 ligand
GDF15: Growth differentiation factor 15
GM-CSF: Granulocyte-macrophage colony-stimulating factor
HE4: Human Epididymis Protein 4
Ig: Immunoglobulin
IL1RA: Interleukin-1 receptor antagonist
IL2: Interleukin 2
IL4: Interleukin 4
IL12: Interleukin 12
IMiD: Immunomodulatory drug
KLK6: Kallikrein-6
kSVM: Extended Support Vector Machine
MIA: Melanoma-derived growth regulatory protein
MGUS: Monoclonal gammopathy of undetermined significance
MM: Multiple myeloma
MYD88: Myeloid differentiation primary response 88
OPG: Osteoprotegerin
PEA: Proximity extension immunoassay
PGF: Placental growth factor
PR: Partial remission
PRL: Prolactin
REG4: Regenerating islet-derived protein 4
SMM: Smoldering multiple myeloma
TGFB1: Transforming growth factor beta 1
THPO: Thrombopoietin
TRAP: Tartrate-resistant acid phosphatase
sAREG: soluble Amphiregulin
sBAFF: soluble B-cell activating factor
sBTC: soluble Betacellulin
sCAIX: soluble Carbonic anhydrase 9
sCD30L: soluble CD30 ligand
sE selectin: soluble E-selectin
sEGFR: soluble Epidermal growth factor receptor
sEMMPRIN: soluble Extracellular matrix metalloproteinase inducer
sEpCAM: soluble Epithelial cell adhesion molecule
sEpiregulin: soluble Epiregulin
sER: soluble Estrogen receptor
sFasL: soluble Fas ligand
sFOLR1: soluble Folate receptor alpha
sHBEGF: soluble Heparin-binding EGF-like growth factor
sHER3: soluble Receptor tyrosine-protein kinase erbB-3
sHER4: soluble Receptor tyrosine-protein kinase erbB-4
sHGF: soluble Hepatocyte growth factor/scatter factor
sHGFR: soluble Hepatocyte growth factor receptor
sIL2RA: soluble Interleukin-2 receptor alpha chain
sIL17RB: soluble Interleukin-17 receptor B
sMICA: soluble MHC class I polypeptide-related sequence A
sPDGFB: soluble Platelet-derived growth factor subunit B
sPECAM1: soluble Platelet endothelial cell adhesion molecule
sSCF: soluble Stem cell factor
sTF: soluble Tissue Factor
sTGFA: soluble Transforming growth factor alpha
sTIE2: soluble Receptor tyrosine kinase Tie2
sTNFR1: soluble Tumor necrosis factor receptor 1
sTNFR2: soluble Tumor necrosis factor receptor 2
sTNFRSF4: soluble Tumor necrosis factor receptor superfamily member 4
suPAR: soluble Urokinase plasminogen activator receptore
sVEGFA: soluble Vascular endothelial growth factor A
VGPR: Very good partial remission
