# Supplementary material for: Serum protein fingerprinting by PEA immunoassay coupled with a pattern-recognition algorithms distinguishes MGUS and multiple myeloma
Source: Oncotarget. 2016 Aug 12;8(41):69408–21. doi: 10.18632/oncotarget.11242 (PMC5642488; doi:10.18632/oncotarget.11242)
Supplement: Supplementary file 1 [file oncotarget-08-69408-s001.pdf]

Serum protein fingerprinting by PEA immunoassay coupled with a pattern-recognition algorithms distinguishes MGUS and multiple myeloma

SUPPLEMENTARY FIGURE AND TABLE

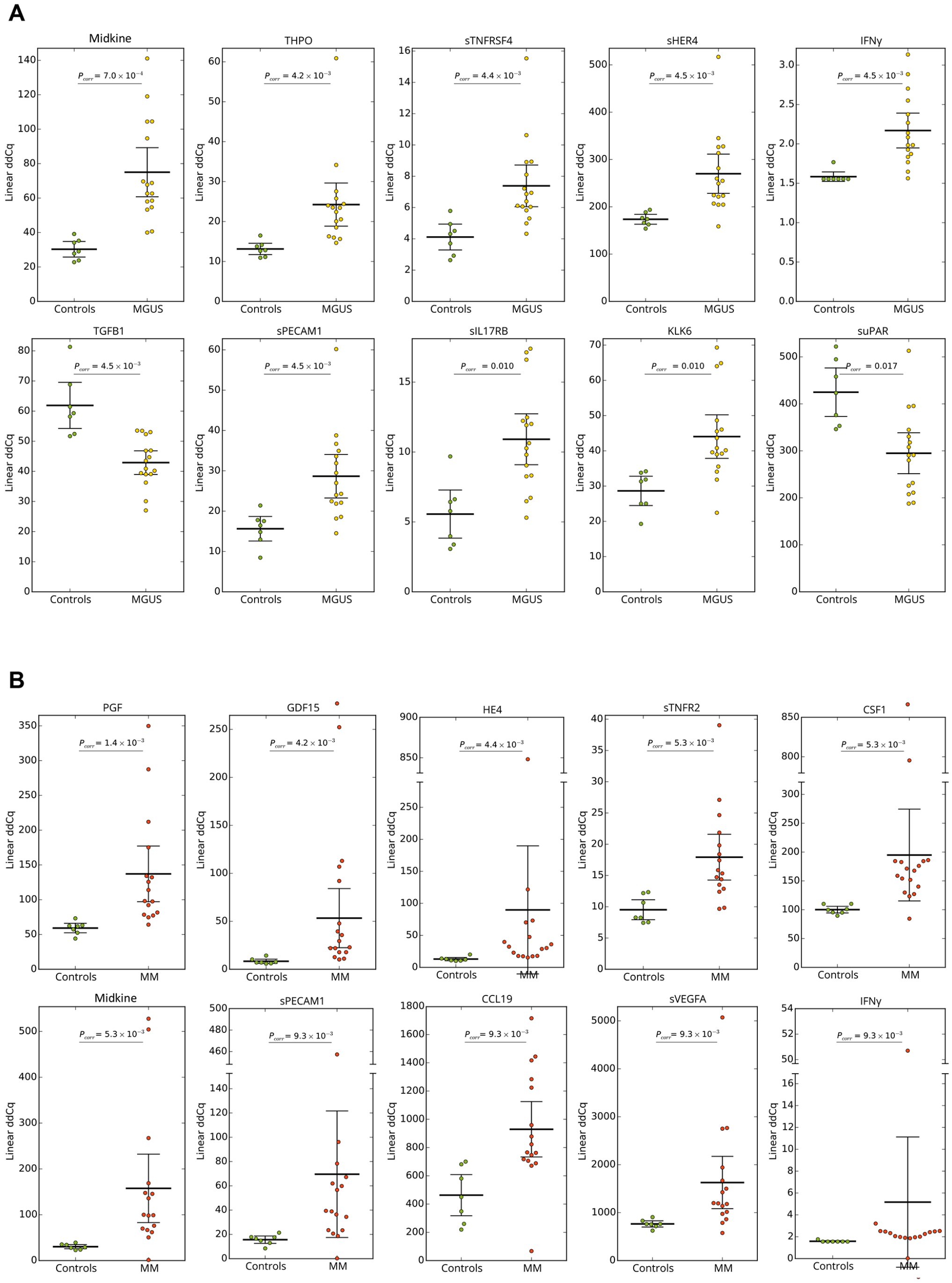

Supplementary Figure S1: The most significant alterations in serum levels of 10 top-ranked proteins between A. healthy controls and MGUS, **B.** healthy controls and MM, and. (*Continued*)

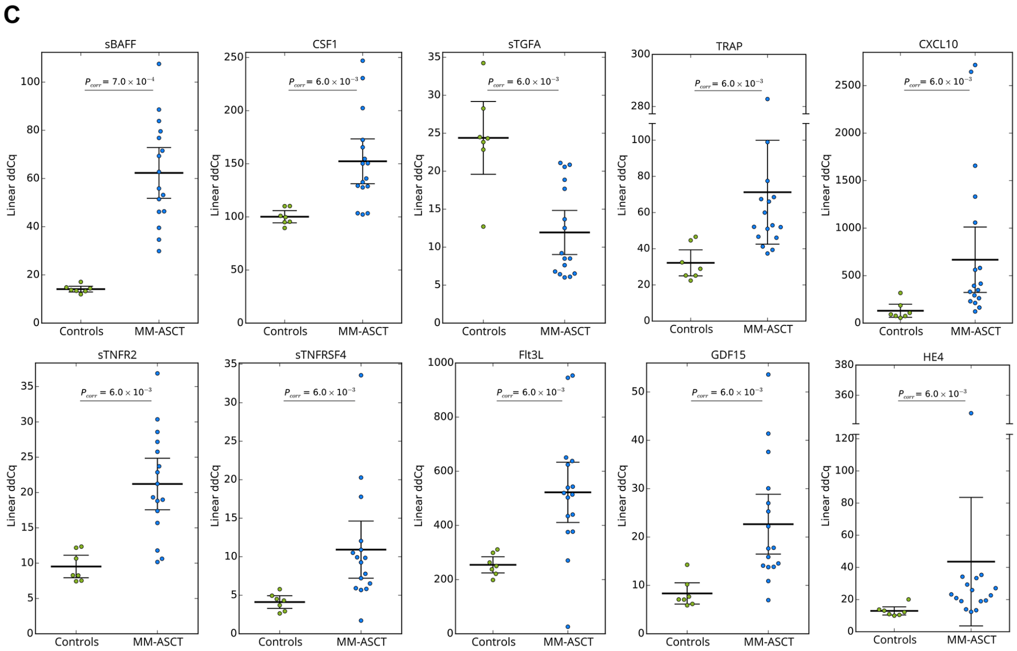

Supplementary Figure S1: The most significant alterations in serum levels of 10 top-ranked proteins between C. healthy controls and MM-ASCT. Group means are indicated by horizontal bars, error bars indicate 95%CI; P_{corr} values for differences between two groups of patients after multiple corrections are stated.

Supplementary Table S1: List of investigated proteins.

See Supplementary File 1

Supplementary Table S2: Serum levels of studied analytes in following patient subgroups: A) MGUS *vs* MM, B) Controls *vs* MGUS, C) Controls *vs* MM, D) MM *vs* MM-ASCT, E) Controls *vs* MM-ASCT.

See Supplementary File 2
