# Supplementary material for: Serum protein fingerprinting by PEA immunoassay coupled with a pattern-recognition algorithms distinguishes MGUS and multiple myeloma
Source: Oncotarget. 2016 Aug 12;8(41):69408–21. doi: 10.18632/oncotarget.11242 (PMC5642488; doi:10.18632/oncotarget.11242)
Supplement: Supplementary file 3 [file oncotarget-08-69408-s003.docx]

**Table S2. Serum levels of studied analytes in following patient subgroups:** A) MGUS *vs* MM, B) Controls *vs* MGUS, C) Controls *vs* MM, D) MM *vs* MM-ASCT, E) Controls *vs* MM-ASCT.

| A) MGUS *vs* MM | | | | | |
| --- | --- | --- | --- | --- | --- |
| *Analyte* | *Mean Linear ddCq (95% CI)* | | *FC* | *P* | *P_corr_* |
|  | *MGUS* | *MM* |  |  |  |
| ADM | 46.9 (28.4-65.4) | 191 (121-262) | 2.80 | 4.0 × 10^-6^ | 3.5 × 10^-4^ |
| TRAP | 28.9 (22.3-35.6) | 85.1 (42.8-127) | 2.33 | 3.7 × 10^-5^ | 1.6 × 10^-3^ |
| GDF15 | 12.6 (9.19-16.0) | 53.3 (22.5-84.1) | 2.72 | 1.1 × 10^-4^ | 3.0 × 10^-3^ |
| suPAR | 295 (251-338) | 486 (368-603) | 1.60 | 2.7 × 10^-4^ | 5.9 × 10^-3^ |
| REG4 | 5.96 (5.46-6.46) | 9.58 (6.78-12.4) | 1.30 | 1.2 × 10^-3^ | .021 |
| TGFB1 | 42.9 (39.0-46.8) | 85.5 (43.7-127) | 1.41 | 1.9 × 10^-3^ | .023 |
| sMICA | 15.4 (9.98-20.8) | 39.4 (26.8-52.1) | 2.29 | 2.1 × 10^-3^ | .023 |
| IL1RA | 9.22 (7.23-11.2) | 60.0 (0-145) | 1.71 | 2.6 × 10^-3^ | .023 |
| HE4 | 18.4 (15.6-21.3) | 89.8 (0-190) | 1.72 | 3.0 × 10^-3^ | .023 |
| sHGFR | 259 (240-278) | 580 (199-960) | 1.26 | 3.0 × 10^-3^ | .023 |
| sVEGFA | 892 (728-1056) | 1628 (1082-2173) | 1.47 | 3.0 × 10^-3^ | .023 |
| sCD30L | 3.40 (2.82-3.98) | 11.4 (2.13-20.7) | 1.76 | 4.5 × 10^-3^ | .032 |
| CCL19 | 606 (481-731) | 929 (733-1125) | 1.34 | 5.8 × 10^-3^ | .038 |
| CSF1 | 130 (116-143) | 195 (115-275) | 1.25 | 8.5 × 10^-3^ | .052 |
| sEpiregulin | 4.73 (3.66-5.80) | 3.85 (2.48-5.23) | .78 | 9.3 × 10^-3^ | .053 |
| MIA | 7.75 (6.97-8.54) | 12.1 (7.71-16.4) | 1.23 | .015 | .082 |
| sCAIX | 6.29 (4.17-8.42) | 3.80 (3.03-4.56) | .72 | .022 | .112 |
| sTNFR1 | 1579 (1345-1814) | 2222 (1605-2840) | 1.33 | .023 | .112 |
| IL12 | 100 (70.9-130) | 172 (118-226) | 1.81 | .026 | .118 |
| Midkine | 75.0 (60.8-89.3) | 158 (82.9-232) | 1.53 | .032 | .137 |
| sIL2RA | 1004 (897-1111) | 1835 (871-2799) | 1.23 | .035 | .137 |
| PGF | 86.7 (73.3-100) | 137 (97.2-177) | 1.28 | .035 | .137 |
| sPECAM1 | 28.7 (23.3-34.1) | 69.6 (17.4-122) | 1.53 | .039 | .145 |
| sEGFR | 25.6 (20.6-30.5) | 22.3 (14.8-29.9) | .77 | .043 | .147 |
| KLK11 | 16.4 (13.2-19.7) | 37.0 (6.91-67.2) | 1.39 | .043 | .147 |
| hGH | 472 (235-709) | 1602 (513-2691) | 2.89 | .047 | .155 |
| CXCL10 | 176 (131-222) | 410 (168-652) | 1.90 | .056 | .173 |
| OPG | 1304 (1065-1543) | 1932 (1315-2549) | 1.44 | .056 | .173 |
| sVEGFD | 181 (160-203) | 255 (164-346) | 1.43 | .061 | .182 |
| sCD69 | 288 (174-402) | 159 (115-202) | .58 | .067 | .192 |
| TNFα | 1.61 (1.45-1.77) | 1.51 (1.44-1.58) | 1.00 | .086 | .239 |
| CXCL9 | 69.3 (55.5-83.2) | 113 (64.4-162) | 1.18 | .110 | .296 |
| FABP4 | 25.3 (16.4-34.3) | 39.4 (22.6-56.1) | 1.35 | .128 | .334 |
| IL6 | 44.0 (29.3-58.8) | 85.9 (48.4-123) | 1.29 | .171 | .405 |
| sTNFR2 | 14.8 (11.5-18.2) | 17.9 (14.3-21.6) | 1.11 | .171 | .405 |
| CA 125 | 3.20 (2.30-4.09) | 7.78 (2.05-13.5) | 1.23 | .179 | .405 |
| sHER2/neu | 105 (91.3-118) | 86.3 (67.9-105) | .87 | .184 | .405 |
| sHER3 | 273 (241-305) | 390 (247-532) | 1.08 | .184 | .405 |
| IL7 | 12.0 (10.6-13.3) | 14.1 (5.78-22.4) | .81 | .184 | .405 |
| PRSS8 | 134 (116-151) | 177 (119-235) | 1.30 | .210 | .451 |
| CASP3 | 151 (116-186) | 181 (34.3-328) | .67 | .224 | .459 |
| sFasL | 3.50 (3.00-4.00) | 5.87 (2.49-9.25) | 1.04 | .224 | .459 |
| sCD40L | 621 (338-905) | 938 (606-1271) | 1.00 | .254 | .508 |
| MYD88 | 9.98 (9.26-10.7) | 9.37 (9.35-9.39) | 1.00 | .281 | .548 |
| EPO | 5.23 (.55-9.92) | 3.66 (1.86-5.46) | 1.09 | .300 | .573 |
| CCL21 | 253 (218-288) | 282 (222-343) | 1.04 | .361 | .664 |
| Cystatin B | 120 (92.5-148) | 140 (101-180) | 1.25 | .381 | .664 |
| sEMMPRIN | 501 (446-556) | 682 (362-1002) | 1.07 | .381 | .664 |
| sTF | 29.5 (25.8-33.2) | 32.6 (26.8-38.4) | 1.07 | .381 | .664 |
| sAREG | 11.6 (9.00-14.2) | 19.0 (5.32-32.8) | 1.14 | .402 | .664 |
| CXCL5 | 420 (344-497) | 534 (390-677) | 1.15 | .402 | .664 |
| sEGF | 446 (345-547) | 389 (282-497) | .79 | .402 | .664 |
| IFNγ | 2.17 (1.95-2.39) | 5.17 (0-11.1) | 1.13 | .423 | .686 |
| THPO | 24.2 (18.8-29.6) | 43.6 (4.60-82.6) | 1.08 | .445 | .709 |
| sHER4 | 270 (228-311) | 245 (185-305) | .93 | .468 | .718 |
| sFOLR1 | 7.92 (6.68-9.15) | 8.95 (6.83-11.1) | 1.14 | .468 | .718 |
| CXCL13 | 311 (236-386) | 418 (250-586) | 1.08 | .491 | .726 |
| sHGF | 39.7 (30.7-48.7) | 47.1 (32.5-61.6) | 1.06 | .515 | .726 |
| sIL6R | 132 (112-152) | 174 (99.3-249) | 1.05 | .515 | .726 |
| TNFSF14 | 23.2 (18.5-27.8) | 27.5 (11.0-44.1) | .80 | .515 | .726 |
| sVEGFR2 | 15.4 (13.7-17.1) | 31.8 (2.10-61.4) | 1.14 | .515 | .726 |
| PSA | 5.89 (2.63-9.15) | 41.5 (0-111) | .50 | .555 | .758 |
| CCL24 | 327 (278-376) | 532 (184-881) | .78 | .564 | .758 |
| CEA | 3.77 (2.80-4.74) | 7.05 (0-15.2) | .79 | .564 | .758 |
| CXCL11 | 48.1 (34.5-61.7) | 77.7 (36.4-119) | 1.07 | .590 | .767 |
| Flt3L | 376 (311-442) | 415 (325-505) | 1.31 | .590 | .767 |
| sE selectin | 43.6 (23.8-63.4) | 43.0 (17.4-68.6) | .94 | .616 | .767 |
| Galectin 3 | 11.7 (9.14-14.3) | 18.4 (3.28-33.6) | 1.01 | .616 | .767 |
| sIL17RB | 10.9 (9.09-12.7) | 12.1 (8.79-15.3) | .98 | .616 | .767 |
| MMP3 | 1.40 (1.29-1.51) | 4.05 (.03-8.08) | 1.04 | .637 | .778 |
| PRL | 25.5 (18.2-32.9) | 47.2 (7.07-87.3) | 1.20 | .642 | .778 |
| sTNFRSF4 | 7.39 (6.05-8.72) | 17.2 (0-37.2) | .97 | .669 | .798 |
| KLK6 | 44.0 (37.9-50.2) | 51.9 (40.3-63.4) | 1.21 | .696 | .798 |
| sPDGFB | 444 (354-534) | 544 (299-789) | 1.11 | .696 | .798 |
| sSCF | 64.6 (58.7-70.4) | 99.9 (45.5-154) | 1.05 | .696 | .798 |
| MPO | 44.4 (34.2-54.7) | 42.8 (32.3-53.3) | 1.02 | .724 | .809 |
| sTGFA | 16.1 (11.2-21.1) | 17.5 (9.90-25.1) | 1.03 | .724 | .809 |
| sFas | 178 (151-205) | 210 (136-284) | 1.04 | .780 | .860 |
| Follistatin | 40.8 (32.5-49.1) | 47.1 (27.9-66.3) | 1.05 | .809 | .879 |
| sBAFF | 19.2 (11.7-26.7) | 18.4 (10.5-26.3) | .89 | .838 | .879 |
| sEpCAM | 3886 (3023-4750) | 4035 (2878-5192) | 1.09 | .838 | .879 |
| IL8 | 297 (202-392) | 294 (191-397) | 1.04 | .838 | .879 |
| sCathepsin D | 9.86 (7.95-11.8) | 10.8 (6.80-14.8) | 1.01 | .926 | .948 |
| CCL2/MCP1 | 2255 (1922-2588) | 2400 (1899-2901) | .94 | .926 | .948 |
| sTIE2 | 26.9 (22.4-31.4) | 27.8 (22.9-32.7) | 1.07 | .956 | .967 |
| sHBEGF | 59.1 (50.6-67.5) | 62.4 (46.0-78.9) | 1.06 | .985 | .985 |

| B) Controls *vs* MGUS | | | | | |
| --- | --- | --- | --- | --- | --- |
| *Analyte* | *Mean Linear ddCq (95% CI)* | | *FC* | *P* | *P_corr_* |
|  | *healthy controls* | *MGUS* |  |  |  |
| Midkine | 30.3 (25.8-34.8) | 75.0 (60.8-89.3) | 2.24 | 8.2 × 10^-6^ | 7.0 × 10^-4^ |
| THPO | 13.1 (11.7-14.6) | 24.2 (18.8-29.6) | 1.79 | 9.8 × 10^-5^ | 4.2 × 10^-3^ |
| sTNFRSF4 | 4.12 (3.29-4.94) | 7.39 (6.05-8.72) | 1.54 | 1.6 × 10^-4^ | 4.4 × 10^-3^ |
| sHER4 | 174 (163-184) | 270 (228-311) | 1.45 | 2.4 × 10^-4^ | 4.5 × 10^-3^ |
| IFNγ | 1.58 (1.52-1.64) | 2.17 (1.95-2.39) | 1.31 | 3.1 × 10^-4^ | 4.5 × 10^-3^ |
| TGFB1 | 61.9 (54.2-69.5) | 42.9 (39.0-46.8) | .71 | 3.7 × 10^-4^ | 4.5 × 10^-3^ |
| sPECAM1 | 15.6 (12.6-18.7) | 28.7 (23.3-34.1) | 1.56 | 3.7 × 10^-4^ | 4.5 × 10^-3^ |
| sIL17RB | 5.56 (3.84-7.28) | 10.9 (9.09-12.7) | 1.81 | 1.1 × 10^-3^ | .010 |
| KLK6 | 28.6 (24.5-32.8) | 44.0 (37.9-50.2) | 1.29 | 1.1 × 10^-3^ | .010 |
| suPAR | 425 (373-476) | 295 (251-338) | .69 | 2.0 × 10^-3^ | .017 |
| sAREG | 6.54 (3.27-9.82) | 11.6 (9.00-14.2) | 1.97 | 2.7 × 10^-3^ | .019 |
| CSF1 | 100 (94.4-106) | 130 (116-143) | 1.31 | 2.7 × 10^-3^ | .019 |
| Cystatin B | 66.0 (49.9-82.1) | 120 (92.5-148) | 1.50 | 3.5 × 10^-3^ | .022 |
| sTIE2 | 17.2 (13.9-20.4) | 26.9 (22.4-31.4) | 1.68 | 3.5 × 10^-3^ | .022 |
| sCAIX | 3.31 (2.83-3.78) | 6.29 (4.17-8.42) | 1.71 | 7.6 × 10^-3^ | .038 |
| IL6 | 18.7 (14.3-23.1) | 44.0 (29.3-58.8) | 1.94 | 7.6 × 10^-3^ | .038 |
| PGF | 59.2 (52.5-66.0) | 86.7 (73.3-100) | 1.35 | 7.6 × 10^-3^ | .038 |
| sEMMPRIN | 387 (362-411) | 501 (446-556) | 1.31 | 9.6 × 10^-3^ | .046 |
| CEA | 1.72 (1.44-2.01) | 3.77 (2.80-4.74) | 2.16 | .010 | .046 |
| Galectin 3 | 7.51 (6.43-8.59) | 11.7 (9.14-14.3) | 1.61 | .012 | .049 |
| sTF | 21.6 (18.6-24.7) | 29.5 (25.8-33.2) | 1.46 | .012 | .049 |
| sFOLR1 | 5.75 (4.83-6.67) | 7.92 (6.68-9.15) | 1.32 | .015 | .058 |
| EPO | 1.40 (1.26-1.54) | 5.23 (.55-9.92) | 1.91 | .017 | .063 |
| IL12 | 54.7 (39.0-70.4) | 100 (70.9-130) | 1.61 | .018 | .066 |
| CCL21 | 194 (173-216) | 253 (218-288) | 1.35 | .022 | .074 |
| HE4 | 12.9 (10.4-15.5) | 18.4 (15.6-21.3) | 1.49 | .022 | .074 |
| CXCL11 | 25.3 (17.9-32.6) | 48.1 (34.5-61.7) | 1.92 | .027 | .081 |
| Follistatin | 28.5 (24.8-32.2) | 40.8 (32.5-49.1) | 1.25 | .027 | .081 |
| sTNFR2 | 9.52 (7.93-11.1) | 14.8 (11.5-18.2) | 1.69 | .027 | .081 |
| sEpCAM | 2684 (1803-3565) | 3886 (3023-4750) | 1.45 | .033 | .086 |
| Flt3L | 254 (225-284) | 376 (311-442) | 1.44 | .033 | .086 |
| sIL6R | 92.5 (81.4-104) | 132 (112-152) | 1.63 | .033 | .086 |
| sTGFA | 24.4 (19.6-29.2) | 16.1 (11.2-21.1) | .55 | .033 | .086 |
| sCD30L | 4.26 (3.77-4.75) | 3.40 (2.82-3.98) | .66 | .055 | .140 |
| sEpiregulin | 3.24 (2.89-3.59) | 4.73 (3.66-5.80) | 1.29 | .065 | .148 |
| CXCL10 | 130 (61.2-199) | 176 (131-222) | 1.55 | .065 | .148 |
| CXCL13 | 187 (148-227) | 311 (236-386) | 1.37 | .065 | .148 |
| sHBEGF | 45.8 (38.7-52.9) | 59.1 (50.6-67.5) | 1.28 | .065 | .148 |
| sCD40L | 1108 (734-1483) | 621 (338-905) | .55 | .076 | .160 |
| CXCL5 | 288 (216-361) | 420 (344-497) | 1.63 | .076 | .160 |
| sHER3 | 228 (201-255) | 273 (241-305) | 1.25 | .076 | .160 |
| sEGFR | 19.2 (16.4-22.0) | 25.6 (20.6-30.5) | 1.23 | .089 | .177 |
| sHGF | 26.9 (22.8-31.1) | 39.7 (30.7-48.7) | 1.41 | .089 | .177 |
| FABP4 | 14.7 (7.91-21.4) | 25.3 (16.4-34.3) | 1.84 | .103 | .196 |
| GDF15 | 8.34 (6.14-10.5) | 12.6 (9.19-16.0) | 1.36 | .103 | .196 |
| TNFα | 1.47 (1.47-1.47) | 1.61 (1.45-1.77) | 1.00 | .116 | .203 |
| sHER2/neu | 81.5 (61.7-101) | 105 (91.3-118) | 1.42 | .118 | .203 |
| sFas | 462 (0-1127) | 178 (151-205) | 1.47 | .118 | .203 |
| sFasL | 2.88 (2.47-3.29) | 3.50 (3.00-4.00) | 1.25 | .118 | .203 |
| REG4 | 5.01 (4.32-5.71) | 5.96 (5.46-6.46) | 1.22 | .118 | .203 |
| CXCL9 | 52.8 (20.9-84.7) | 69.3 (55.5-83.2) | 2.05 | .135 | .224 |
| CASP3 | 90.3 (64.5-116) | 151 (116-186) | 1.43 | .135 | .224 |
| CA 125 | 2.08 (1.63-2.52) | 3.20 (2.30-4.09) | 1.47 | .139 | .225 |
| MYD88 | 9.36 (9.36-9.36) | 9.98 (9.26-10.7) | 1.00 | .172 | .269 |
| sCD69 | 154 (96.9-210) | 288 (174-402) | 1.48 | .175 | .269 |
| IL1RA | 12.1 (9.02-15.2) | 9.22 (7.23-11.2) | .76 | .175 | .269 |
| sVEGFR2 | 13.8 (12.5-15.0) | 15.4 (13.7-17.1) | 1.11 | .198 | .298 |
| ADM | 65.0 (47.5-82.4) | 46.9 (28.4-65.4) | .84 | .222 | .313 |
| sCathepsin D | 7.52 (5.44-9.60) | 9.86 (7.95-11.8) | 1.13 | .222 | .313 |
| sE selectin | 24.9 (15.0-34.8) | 43.6 (23.8-63.4) | 2.07 | .222 | .313 |
| PRSS8 | 118 (95.4-140) | 134 (116-151) | 1.22 | .222 | .313 |
| CCL19 | 463 (317-609) | 606 (481-731) | 1.31 | .249 | .340 |
| sVEGFA | 765 (700-830) | 892 (728-1056) | 1.07 | .249 | .340 |
| MIA | 6.69 (5.55-7.83) | 7.75 (6.97-8.54) | 1.15 | .278 | .367 |
| PRL | 17.8 (14.0-21.5) | 25.5 (18.2-32.9) | 1.27 | .278 | .367 |
| sSCF | 58.6 (50.1-67.0) | 64.6 (58.7-70.4) | 1.08 | .308 | .402 |
| sMICA | 10.4 (5.91-14.9) | 15.4 (9.98-20.8) | 1.51 | .332 | .426 |
| IL7 | 10.8 (8.86-12.8) | 12.0 (10.6-13.3) | 1.10 | .341 | .431 |
| CCL24 | 302 (210-393) | 327 (278-376) | 1.18 | .376 | .462 |
| OPG | 1086 (940-1231) | 1304 (1065-1543) | 1.11 | .376 | .462 |
| sIL2RA | 906 (770-1042) | 1004 (897-1111) | 1.09 | .413 | .500 |
| IL8 | 215 (178-253) | 297 (202-392) | 1.07 | .535 | .630 |
| sPDGFB | 381 (339-423) | 444 (354-534) | 1.05 | .535 | .630 |
| sBAFF | 14.1 (12.9-15.3) | 19.2 (11.7-26.7) | 1.11 | .624 | .716 |
| sVEGFD | 170 (149-191) | 181 (160-203) | .99 | .624 | .716 |
| sEGF | 392 (219-564) | 446 (345-547) | 1.19 | .671 | .760 |
| sTNFR1 | 1496 (1313-1679) | 1579 (1345-1814) | 1.18 | .720 | .794 |
| TRAP | 32.2 (25.0-39.4) | 28.9 (22.3-35.6) | .96 | .720 | .794 |
| MMP3 | 1.39 (1.28-1.49) | 1.40 (1.29-1.51) | .98 | .815 | .887 |
| PSA | 4.32 (2.24-6.40) | 5.89 (2.63-9.15) | 1.15 | .838 | .901 |
| MPO | 49.4 (23.0-75.7) | 44.4 (34.2-54.7) | 1.01 | .871 | .913 |
| TNFSF14 | 22.0 (15.5-28.4) | 23.2 (18.5-27.8) | 1.22 | .871 | .913 |
| KLK11 | 15.5 (12.6-18.4) | 16.4 (13.2-19.7) | 1.02 | .922 | .944 |
| CCL2/MCP1 | 2133 (1721-2544) | 2255 (1922-2588) | 1.17 | .922 | .944 |
| hGH | 601 (193-1009) | 472 (235-709) | .43 | .974 | .974 |
| sHGFR | 254 (236-272) | 259 (240-278) | 1.07 | .974 | .974 |

| C) Controls *vs* MM | | | | | |
| --- | --- | --- | --- | --- | --- |
| *Analyte* | *Mean Linear ddCq (95% CI)* | | *FC* | *P* | *P_corr_* |
|  |  | |  |  |  |
|  | *healthy controls* | *MM* |  |  |  |
| PGF | 59.2 (52.5-66.0) | 137 (97.2-177) | 1.72 | 1.6 × 10^-5^ | 1.4 × 10^-3^ |
| GDF15 | 8.34 (6.14-10.5) | 53.3 (22.5-84.1) | 3.69 | 9.8 × 10^-5^ | 4.2 × 10^-3^ |
| HE4 | 12.9 (10.4-15.5) | 89.8 (0-190) | 2.56 | 1.6 × 10^-4^ | 4.4 × 10^-3^ |
| sTNFR2 | 9.52 (7.93-11.1) | 17.9 (14.3-21.6) | 1.88 | 2.4 × 10^-4^ | 5.3 × 10^-3^ |
| CSF1 | 100 (94.4-106) | 195 (115-275) | 1.64 | 3.7 × 10^-4^ | 5.3 × 10^-3^ |
| Midkine | 30.3 (25.8-34.8) | 158 (82.9-232) | 3.42 | 3.7 × 10^-4^ | 5.3 × 10^-3^ |
| sPECAM1 | 15.6 (12.6-18.7) | 69.6 (17.4-122) | 2.38 | 7.7 × 10^-4^ | 9.3 × 10^-3^ |
| CCL19 | 463 (317-609) | 929 (733-1125) | 1.76 | 1.1 × 10^-3^ | 9.3 × 10^-3^ |
| sVEGFA | 765 (700-830) | 1628 (1082-2173) | 1.57 | 1.1 × 10^-3^ | 9.3 × 10^-3^ |
| IFNγ | 1.58 (1.52-1.64) | 5.17 (0-11.1) | 1.47 | 1.1 × 10^-3^ | 9.3 × 10^-3^ |
| REG4 | 5.01 (4.32-5.71) | 9.58 (6.78-12.4) | 1.59 | 1.5 × 10^-3^ | .011 |
| sEMMPRIN | 387 (362-411) | 682 (362-1002) | 1.40 | 2.0 × 10^-3^ | .011 |
| Galectin 3 | 7.51 (6.43-8.59) | 18.4 (3.28-33.6) | 1.63 | 2.0 × 10^-3^ | .011 |
| IL12 | 54.7 (39.0-70.4) | 172 (118-226) | 2.92 | 2.0 × 10^-3^ | .011 |
| sMICA | 10.4 (5.91-14.9) | 39.4 (26.8-52.1) | 3.46 | 2.0 × 10^-3^ | .011 |
| TRAP | 32.2 (25.0-39.4) | 85.1 (42.8-127) | 2.23 | 2.0 × 10^-3^ | .011 |
| EPO | 1.40 (1.26-1.54) | 3.66 (1.86-5.46) | 2.07 | 2.3 × 10^-3^ | .012 |
| ADM | 65.0 (47.5-82.4) | 191 (121-262) | 2.35 | 2.7 × 10^-3^ | .012 |
| IL6 | 18.7 (14.3-23.1) | 85.9 (48.4-123) | 2.51 | 2.7 × 10^-3^ | .012 |
| KLK6 | 28.6 (24.5-32.8) | 51.9 (40.3-63.4) | 1.57 | 2.7 × 10^-3^ | .012 |
| THPO | 13.1 (11.7-14.6) | 43.6 (4.60-82.6) | 1.93 | 3.5 × 10^-3^ | .014 |
| MIA | 6.69 (5.55-7.83) | 12.1 (7.71-16.4) | 1.42 | 4.6 × 10^-3^ | .018 |
| sAREG | 6.54 (3.27-9.82) | 19.0 (5.32-32.8) | 2.24 | 5.9 × 10^-3^ | .019 |
| Cystatin B | 66.0 (49.9-82.1) | 140 (101-180) | 1.87 | 5.9 × 10^-3^ | .019 |
| sHGF | 26.9 (22.8-31.1) | 47.1 (32.5-61.6) | 1.50 | 5.9 × 10^-3^ | .019 |
| sTGFA | 24.4 (19.6-29.2) | 17.5 (9.90-25.1) | .57 | 5.9 × 10^-3^ | .019 |
| sTIE2 | 17.2 (13.9-20.4) | 27.8 (22.9-32.7) | 1.79 | 5.9 × 10^-3^ | .019 |
| CCL21 | 194 (173-216) | 282 (222-343) | 1.40 | 7.6 × 10^-3^ | .022 |
| sTF | 21.6 (18.6-24.7) | 32.6 (26.8-38.4) | 1.56 | 7.6 × 10^-3^ | .022 |
| CXCL13 | 187 (148-227) | 418 (250-586) | 1.48 | 9.6 × 10^-3^ | .026 |
| FABP4 | 14.7 (7.91-21.4) | 39.4 (22.6-56.1) | 2.49 | 9.6 × 10^-3^ | .026 |
| sTNFRSF4 | 4.12 (3.29-4.94) | 17.2 (0-37.2) | 1.49 | 9.6 × 10^-3^ | .026 |
| CEA | 1.72 (1.44-2.01) | 7.05 (0-15.2) | 1.70 | .010 | .026 |
| CA125 | 2.08 (1.63-2.52) | 7.78 (2.05-13.5) | 1.81 | .011 | .029 |
| sHGFR | 254 (236-272) | 580 (199-960) | 1.34 | .012 | .029 |
| sIL2RA | 906 (770-1042) | 1835 (871-2799) | 1.35 | .012 | .029 |
| CXCL10 | 130 (61.2-199) | 410 (168-652) | 2.94 | .015 | .031 |
| sHER3 | 228 (201-255) | 390 (247-532) | 1.35 | .015 | .031 |
| sFOLR1 | 5.75 (4.83-6.67) | 8.95 (6.83-11.1) | 1.50 | .015 | .031 |
| sIL17RB | 5.56 (3.84-7.28) | 12.1 (8.79-15.3) | 1.78 | .015 | .031 |
| OPG | 1086 (940-1231) | 1932 (1315-2549) | 1.60 | .015 | .031 |
| Flt3L | 254 (225-284) | 415 (325-505) | 1.88 | .027 | .056 |
| CXCL5 | 288 (216-361) | 534 (390-677) | 1.89 | .033 | .066 |
| CXCL9 | 52.8 (20.9-84.7) | 113 (64.4-162) | 2.41 | .039 | .075 |
| sFasL | 2.88 (2.47-3.29) | 5.87 (2.49-9.25) | 1.30 | .039 | .075 |
| sTNFR1 | 1496 (1313-1679) | 2222 (1605-2840) | 1.58 | .047 | .088 |
| sIL6R | 92.5 (81.4-104) | 174 (99.3-249) | 1.71 | .055 | .099 |
| PRSS8 | 118 (95.4-140) | 177 (119-235) | 1.59 | .055 | .099 |
| CXCL11 | 25.3 (17.9-32.6) | 77.7 (36.4-119) | 2.05 | .076 | .134 |
| KLK11 | 15.5 (12.6-18.4) | 37.0 (6.91-67.2) | 1.42 | .089 | .150 |
| sVEGFD | 170 (149-191) | 255 (164-346) | 1.42 | .089 | .150 |
| Follistatin | 28.5 (24.8-32.2) | 47.1 (27.9-66.3) | 1.31 | .103 | .170 |
| sHER4 | 174 (163-184) | 245 (185-305) | 1.34 | .118 | .192 |
| sEpCAM | 2684 (1803-3565) | 4035 (2878-5192) | 1.58 | .135 | .215 |
| sFas | 462 (0-1127) | 210 (136-284) | 1.53 | .154 | .241 |
| sCathepsin D | 7.52 (5.44-9.60) | 10.8 (6.80-14.8) | 1.13 | .198 | .288 |
| hGH | 601 (193-1009) | 1602 (513-2691) | 1.24 | .198 | .288 |
| IL1RA | 12.1 (9.02-15.2) | 60.0 (0-145) | 1.29 | .198 | .288 |
| sVEGFR2 | 13.8 (12.5-15.0) | 31.8 (2.10-61.4) | 1.26 | .198 | .288 |
| sHBEGF | 45.8 (38.7-52.9) | 62.4 (46.0-78.9) | 1.36 | .222 | .313 |
| PRL | 17.8 (14.0-21.5) | 47.2 (7.07-87.3) | 1.53 | .222 | .313 |
| sE selectin | 24.9 (15.0-34.8) | 43.0 (17.4-68.6) | 1.95 | .278 | .385 |
| sCD30L | 4.26 (3.77-4.75) | 11.4 (2.13-20.7) | 1.16 | .341 | .466 |
| sCAIX | 3.31 (2.83-3.78) | 3.80 (3.03-4.56) | 1.22 | .365 | .490 |
| MYD88 | 9.36 (9.36-9.36) | 9.37 (9.35-9.39) | 1.00 | .374 | .490 |
| sCD40L | 1108 (734-1483) | 938 (606-1271) | .55 | .376 | .490 |
| CASP3 | 90.3 (64.5-116) | 181 (34.3-328) | .96 | .492 | .613 |
| sPDGFB | 381 (339-423) | 544 (299-789) | 1.17 | .492 | .613 |
| sSCF | 58.6 (50.1-67.0) | 99.9 (45.5-154) | 1.14 | .492 | .613 |
| suPAR | 425 (373-476) | 486 (368-603) | 1.10 | .535 | .657 |
| TNFα | 1.47 (1.47-1.47) | 1.51 (1.44-1.58) | 1.00 | .571 | .691 |
| IL8 | 215 (178-253) | 294 (191-397) | 1.11 | .579 | .691 |
| IL7 | 10.8 (8.86-12.8) | 14.1 (5.78-22.4) | .89 | .624 | .736 |
| sEGFR | 19.2 (16.4-22.0) | 22.3 (14.8-29.9) | .95 | .671 | .780 |
| CCL2/MCP1 | 2133 (1721-2544) | 2400 (1899-2901) | 1.09 | .720 | .825 |
| sHER2/neu | 81.5 (61.7-101) | 86.3 (67.9-105) | 1.23 | .769 | .848 |
| TGFB1 | 61.9 (54.2-69.5) | 85.5 (43.7-127) | 1.00 | .769 | .848 |
| TNFSF14 | 22.0 (15.5-28.4) | 27.5 (11.0-44.1) | .97 | .769 | .848 |
| MMP3 | 1.39 (1.28-1.49) | 4.05 (.03-8.08) | 1.02 | .841 | .915 |
| sEpiregulin | 3.24 (2.89-3.59) | 3.85 (2.48-5.23) | 1.00 | .854 | .918 |
| sBAFF | 14.1 (12.9-15.3) | 18.4 (10.5-26.3) | .98 | .871 | .924 |
| sCD69 | 154 (96.9-210) | 159 (115-202) | .86 | .974 | 1.000 |
| MPO | 49.4 (23.0-75.7) | 42.8 (32.3-53.3) | 1.03 | .974 | 1.000 |
| CCL24 | 302 (210-393) | 532 (184-881) | .92 | 1.000 | 1.000 |
| sEGF | 392 (219-564) | 389 (282-497) | .93 | 1.000 | 1.000 |
| PSA | 4.32 (2.24-6.40) | 41.5 (0-111) | .57 | 1.000 | 1.000 |

| D) MM *vs* MM-ASCT | | | | | |
| --- | --- | --- | --- | --- | --- |
| *Analyte* | *Mean Linear ddCq (95% CI)* | | *FC* | *P* | *P_corr_* |
|  | *MM* | *MM-ASCT* |  |  |  |
| REG4 | 9.58 (6.78-12.4) | 7.01 (4.50-9.51) | .74 | 3.1 × 10^-5^ | 2.6 × 10^-3^ |
| sBAFF | 18.4 (10.5-26.3) | 62.3 (51.8-72.9) | 4.38 | 1.5 × 10^-4^ | 6.1 × 10^-3^ |
| sPECAM1 | 69.6 (17.4-122) | 17.3 (13.1-21.6) | .41 | 2.1 × 10^-4^ | 6.1 × 10^-3^ |
| sIL6R | 174 (99.3-249) | 81.6 (64.6-98.6) | .55 | 5.8 × 10^-4^ | .012 |
| sPDGFB | 544 (299-789) | 274 (222-327) | .56 | 7.6 × 10^-4^ | .013 |
| Midkine | 158 (82.9-232) | 54.5 (42.7-66.3) | .54 | 1.0 × 10^-3^ | .014 |
| sHGF | 47.1 (32.5-61.6) | 26.9 (20.7-33.1) | .63 | 1.3 × 10^-3^ | .014 |
| TGFB1 | 85.5 (43.7-127) | 41.6 (34.9-48.3) | .72 | 1.3 × 10^-3^ | .014 |
| sAREG | 19.0 (5.32-32.8) | 7.52 (6.17-8.87) | .67 | 1.7 × 10^-3^ | .016 |
| sMICA | 39.4 (26.8-52.1) | 22.5 (15.8-29.2) | .59 | 2.1 × 10^-3^ | .018 |
| CXCL5 | 534 (390-677) | 340 (252-427) | .66 | 2.7 × 10^-3^ | .019 |
| sVEGFA | 1628 (1082-2173) | 979 (786-1172) | .74 | 2.7 × 10^-3^ | .019 |
| sHBEGF | 62.4 (46.0-78.9) | 35.7 (28.1-43.3) | .46 | 3.4 × 10^-3^ | .022 |
| OPG | 1932 (1315-2549) | 1208 (997-1420) | .74 | 6.3 × 10^-3^ | .036 |
| sTF | 32.6 (26.8-38.4) | 26.6 (22.5-30.7) | .79 | 6.3 × 10^-3^ | .036 |
| CXCL9 | 113 (64.4-162) | 149 (108-189) | 1.89 | 7.6 × 10^-3^ | .041 |
| CXCL13 | 418 (250-586) | 239 (181-297) | .69 | .011 | .055 |
| CA125 | 7.78 (2.05-13.5) | 2.59 (2.19-2.99) | .77 | .011 | .055 |
| CCL21 | 282 (222-343) | 179 (149-209) | .73 | .013 | .056 |
| sE selectin | 43.0 (17.4-68.6) | 21.8 (13.8-29.8) | .50 | .013 | .056 |
| ADM | 191 (121-262) | 98.6 (79.5-118) | .56 | .016 | .063 |
| CCL19 | 929 (733-1125) | 633 (485-781) | .71 | .021 | .084 |
| sHER4 | 245 (185-305) | 176 (143-208) | .76 | .025 | .093 |
| Flt3L | 415 (325-505) | 522 (411-633) | 1.10 | .034 | .120 |
| MYD88 | 9.37 (9.35-9.39) | 9.85 (9.47-10.2) | 1.00 | .044 | .151 |
| sCD40L | 938 (606-1271) | 1260 (980-1539) | 1.88 | .051 | .156 |
| FABP4 | 39.4 (22.6-56.1) | 21.3 (13.7-28.9) | .59 | .051 | .156 |
| IL6 | 85.9 (48.4-123) | 48.0 (23.2-72.8) | .64 | .051 | .156 |
| sEMMPRIN | 682 (362-1002) | 432 (354-509) | .80 | .058 | .171 |
| sTIE2 | 27.8 (22.9-32.7) | 23.3 (20.2-26.5) | .84 | .065 | .181 |
| sTNFRSF4 | 17.2 (0-37.2) | 10.9 (7.21-14.6) | 1.49 | .065 | .181 |
| CXCL10 | 410 (168-652) | 667 (323-1011) | 1.38 | .083 | .217 |
| sHGFR | 580 (199-960) | 474 (87.6-860) | .84 | .083 | .217 |
| PGF | 137 (97.2-177) | 106 (85.6-127) | .95 | .093 | .230 |
| sTGFA | 17.5 (9.90-25.1) | 11.9 (9.03-14.8) | .64 | .093 | .230 |
| Follistatin | 47.1 (27.9-66.3) | 28.8 (22.5-35.0) | .81 | .105 | .250 |
| sCAIX | 3.80 (3.03-4.56) | 4.89 (3.97-5.82) | 1.24 | .117 | .257 |
| CASP3 | 181 (34.3-328) | 164 (113-215) | 1.25 | .117 | .257 |
| sIL17RB | 12.1 (8.79-15.3) | 10.0 (8.25-11.8) | 1.02 | .117 | .257 |
| CCL24 | 532 (184-881) | 558 (0-1254) | .64 | .130 | .279 |
| IL1RA | 60.0 (0-145) | 56.8 (0-147) | .65 | .159 | .334 |
| CSF1 | 195 (115-275) | 152 (131-173) | .88 | .175 | .351 |
| IFNγ | 5.17 (0-11.1) | 2.44 (1.98-2.90) | 1.13 | .175 | .351 |
| sTNFR2 | 17.9 (14.3-21.6) | 21.2 (17.6-24.9) | 1.30 | .193 | .377 |
| GDF15 | 53.3 (22.5-84.1) | 22.7 (16.5-28.8) | .68 | .211 | .395 |
| THPO | 43.6 (4.60-82.6) | 28.5 (21.7-35.3) | 1.15 | .211 | .395 |
| EPO | 3.66 (1.86-5.46) | 2.57 (1.84-3.30) | .82 | .252 | .425 |
| Galectin 3 | 18.4 (3.28-33.6) | 9.56 (7.69-11.4) | .74 | .252 | .425 |
| HE4 | 89.8 (0-190) | 43.6 (3.61-83.5) | .73 | .252 | .425 |
| IL12 | 172 (118-226) | 136 (78.7-193) | .76 | .252 | .425 |
| KLK6 | 51.9 (40.3-63.4) | 44.7 (36.1-53.3) | .79 | .252 | .425 |
| sEGF | 389 (282-497) | 462 (373-550) | 1.21 | .274 | .445 |
| sHER2/neu | 86.3 (67.9-105) | 90.4 (71.9-109) | .98 | .274 | .445 |
| KLK11 | 37.0 (6.91-67.2) | 49.3 (0-110) | .88 | .298 | .474 |
| MMP3 | 4.05 (.03-8.08) | 3.55 (0-7.86) | .94 | .315 | .493 |
| sCD69 | 159 (115-202) | 233 (129-337) | 1.15 | .348 | .517 |
| CXCL11 | 77.7 (36.4-119) | 68.1 (48.1-88.0) | 1.57 | .348 | .517 |
| hGH | 1602 (513-2691) | 948 (475-1420) | .66 | .348 | .517 |
| sCD30L | 11.4 (2.13-20.7) | 8.40 (2.25-14.6) | .94 | .404 | .579 |
| sHER3 | 390 (247-532) | 363 (166-559) | .81 | .404 | .579 |
| CCL2/MCP1 | 2400 (1899-2901) | 2701 (1856-3546) | 1.02 | .433 | .601 |
| sVEGFD | 255 (164-346) | 241 (192-290) | 1.00 | .433 | .601 |
| sIL2RA | 1835 (871-2799) | 1485 (798-2171) | .99 | .495 | .676 |
| PSA | 41.5 (0-111) | 13.8 (0-30.0) | 1.00 | .554 | .743 |
| PRSS8 | 177 (119-235) | 141 (112-170) | .84 | .562 | .743 |
| sSCF | 99.9 (45.5-154) | 142 (4.03-281) | 1.15 | .632 | .821 |
| Cystatin B | 140 (101-180) | 114 (88.1-140) | .83 | .669 | .821 |
| sFas | 210 (136-284) | 186 (156-217) | .92 | .669 | .821 |
| IL7 | 14.1 (5.78-22.4) | 10.9 (9.44-12.3) | 1.06 | .669 | .821 |
| IL8 | 294 (191-397) | 299 (159-440) | .80 | .669 | .821 |
| sEpiregulin | 3.85 (2.48-5.23) | 4.67 (1.56-7.77) | 1.00 | .689 | .835 |
| sVEGFR2 | 31.8 (2.10-61.4) | 29.9 (.11-59.7) | .80 | .706 | .843 |
| suPAR | 486 (368-603) | 417 (337-497) | .95 | .744 | .876 |
| TNFα | 1.51 (1.44-1.58) | 1.46 (1.32-1.60) | 1.00 | .787 | .915 |
| sFOLR1 | 8.95 (6.83-11.1) | 7.84 (6.77-8.91) | 1.01 | .821 | .941 |
| sFasL | 5.87 (2.49-9.25) | 5.91 (1.15-10.7) | 1.01 | .900 | .974 |
| MPO | 42.8 (32.3-53.3) | 39.3 (29.4-49.1) | .96 | .900 | .974 |
| PRL | 47.2 (7.07-87.3) | 28.1 (22.4-33.9) | 1.00 | .900 | .974 |
| TRAP | 85.1 (42.8-127) | 71.2 (42.5-100.0) | .81 | .900 | .974 |
| sCathepsin D | 10.8 (6.80-14.8) | 9.10 (7.03-11.2) | 1.00 | .940 | .974 |
| sEGFR | 22.3 (14.8-29.9) | 24.5 (11.8-37.2) | .96 | .940 | .974 |
| sTNFR1 | 2222 (1605-2840) | 1906 (1511-2301) | .90 | .940 | .974 |
| TNFSF14 | 27.5 (11.0-44.1) | 21.1 (17.0-25.2) | 1.09 | .940 | .974 |
| sEpCAM | 4035 (2878-5192) | 4050 (2823-5277) | .94 | .980 | .980 |
| CEA | 7.05 (0-15.2) | 2.88 (2.30-3.47) | 1.01 | .980 | .980 |
| MIA | 12.1 (7.71-16.4) | 10.6 (9.03-12.2) | 1.02 | .980 | .980 |

| E) Controls *vs* MM-ASCT | | | | | |
| --- | --- | --- | --- | --- | --- |
| *Analyte* | *Mean Linear ddCq (95% CI)* | | *FC* | *P* | *P_corr_* |
|  | *healthy controls* | *MM-ASCT* |  |  |  |
| sBAFF | 14.1 (12.9-15.3) | 62.3 (51.8-72.9) | 4.32 | 8.2 × 10^-6^ | 7.0 × 10^-4^ |
| CSF1 | 100 (94.4-106) | 152 (131-173) | 1.44 | 2.4 × 10^-4^ | 6.0 × 10^-3^ |
| sTGFA | 24.4 (19.6-29.2) | 11.9 (9.03-14.8) | .36 | 2.4 × 10^-4^ | 6.0 × 10^-3^ |
| TRAP | 32.2 (25.0-39.4) | 71.2 (42.5-100.0) | 1.82 | 3.7 × 10^-4^ | 6.0 × 10^-3^ |
| CXCL10 | 130 (61.2-199) | 667 (323-1011) | 4.05 | 5.4 × 10^-4^ | 6.0 × 10^-3^ |
| sTNFR2 | 9.52 (7.93-11.1) | 21.2 (17.6-24.9) | 2.45 | 5.4 × 10^-4^ | 6.0 × 10^-3^ |
| sTNFRSF4 | 4.12 (3.29-4.94) | 10.9 (7.21-14.6) | 2.22 | 5.4 × 10^-4^ | 6.0 × 10^-3^ |
| Flt3L | 254 (225-284) | 522 (411-633) | 2.07 | 7.7 × 10^-4^ | 6.0 × 10^-3^ |
| GDF15 | 8.34 (6.14-10.5) | 22.7 (16.5-28.8) | 2.50 | 7.7 × 10^-4^ | 6.0 × 10^-3^ |
| HE4 | 12.9 (10.4-15.5) | 43.6 (3.61-83.5) | 1.88 | 7.7 × 10^-4^ | 6.0 × 10^-3^ |
| THPO | 13.1 (11.7-14.6) | 28.5 (21.7-35.3) | 2.23 | 7.7 × 10^-4^ | 6.0 × 10^-3^ |
| TGFB1 | 61.9 (54.2-69.5) | 41.6 (34.9-48.3) | .72 | 1.1 × 10^-3^ | 7.1 × 10^-3^ |
| PGF | 59.2 (52.5-66.0) | 106 (85.6-127) | 1.64 | 1.1 × 10^-3^ | 7.1 × 10^-3^ |
| EPO | 1.40 (1.26-1.54) | 2.57 (1.84-3.30) | 1.70 | 1.5 × 10^-3^ | 9.0 × 10^-3^ |
| CXCL9 | 52.8 (20.9-84.7) | 149 (108-189) | 4.56 | 2.7 × 10^-3^ | .015 |
| IFNγ | 1.58 (1.52-1.64) | 2.44 (1.98-2.90) | 1.67 | 3.2 × 10^-3^ | .016 |
| CXCL11 | 25.3 (17.9-32.6) | 68.1 (48.1-88.0) | 3.21 | 3.5 × 10^-3^ | .016 |
| MIA | 6.69 (5.55-7.83) | 10.6 (9.03-12.2) | 1.45 | 3.5 × 10^-3^ | .016 |
| Midkine | 30.3 (25.8-34.8) | 54.5 (42.7-66.3) | 1.84 | 3.5 × 10^-3^ | .016 |
| IL12 | 54.7 (39.0-70.4) | 136 (78.7-193) | 2.23 | 4.6 × 10^-3^ | .019 |
| sIL17RB | 5.56 (3.84-7.28) | 10.0 (8.25-11.8) | 1.80 | 4.6 × 10^-3^ | .019 |
| CEA | 1.72 (1.44-2.01) | 2.88 (2.30-3.47) | 1.72 | 5.5 × 10^-3^ | .022 |
| KLK6 | 28.6 (24.5-32.8) | 44.7 (36.1-53.3) | 1.24 | 5.9 × 10^-3^ | .022 |
| sCAIX | 3.31 (2.83-3.78) | 4.89 (3.97-5.82) | 1.52 | 9.6 × 10^-3^ | .034 |
| sMICA | 10.4 (5.91-14.9) | 22.5 (15.8-29.2) | 2.04 | .011 | .038 |
| IL6 | 18.7 (14.3-23.1) | 48.0 (23.2-72.8) | 1.60 | .012 | .040 |
| Cystatin B | 66.0 (49.9-82.1) | 114 (88.1-140) | 1.56 | .015 | .046 |
| sFOLR1 | 5.75 (4.83-6.67) | 7.84 (6.77-8.91) | 1.51 | .015 | .046 |
| sPDGFB | 381 (339-423) | 274 (222-327) | .65 | .018 | .054 |
| Galectin 3 | 7.51 (6.43-8.59) | 9.56 (7.69-11.4) | 1.21 | .022 | .062 |
| sTIE2 | 17.2 (13.9-20.4) | 23.3 (20.2-26.5) | 1.49 | .022 | .062 |
| ADM | 65.0 (47.5-82.4) | 98.6 (79.5-118) | 1.32 | .047 | .119 |
| PRL | 17.8 (14.0-21.5) | 28.1 (22.4-33.9) | 1.52 | .047 | .119 |
| sSCF | 58.6 (50.1-67.0) | 142 (4.03-281) | 1.31 | .047 | .119 |
| MYD88 | 9.36 (9.36-9.36) | 9.85 (9.47-10.2) | 1.00 | .049 | .121 |
| sIL2RA | 906 (770-1042) | 1485 (798-2171) | 1.33 | .055 | .129 |
| sVEGFD | 170 (149-191) | 241 (192-290) | 1.42 | .055 | .129 |
| sEpCAM | 2684 (1803-3565) | 4050 (2823-5277) | 1.48 | .065 | .148 |
| sHBEGF | 45.8 (38.7-52.9) | 35.7 (28.1-43.3) | .62 | .076 | .168 |
| CASP3 | 90.3 (64.5-116) | 164 (113-215) | 1.20 | .089 | .191 |
| CA 125 | 2.08 (1.63-2.52) | 2.59 (2.19-2.99) | 1.40 | .111 | .231 |
| CCL24 | 302 (210-393) | 558 (0-1254) | .59 | .118 | .231 |
| FABP4 | 14.7 (7.91-21.4) | 21.3 (13.7-28.9) | 1.48 | .118 | .231 |
| sFasL | 2.88 (2.47-3.29) | 5.91 (1.15-10.7) | 1.31 | .118 | .231 |
| sFas | 462 (0-1127) | 186 (156-217) | 1.41 | .135 | .248 |
| sTF | 21.6 (18.6-24.7) | 26.6 (22.5-30.7) | 1.23 | .135 | .248 |
| sVEGFA | 765 (700-830) | 979 (786-1172) | 1.17 | .135 | .248 |
| sAREG | 6.54 (3.27-9.82) | 7.52 (6.17-8.87) | 1.51 | .154 | .276 |
| REG4 | 5.01 (4.32-5.71) | 7.01 (4.50-9.51) | 1.17 | .175 | .307 |
| KLK11 | 15.5 (12.6-18.4) | 49.3 (0-110) | 1.25 | .198 | .340 |
| PRSS8 | 118 (95.4-140) | 141 (112-170) | 1.33 | .222 | .368 |
| sTNFR1 | 1496 (1313-1679) | 1906 (1511-2301) | 1.42 | .222 | .368 |
| CCL19 | 463 (317-609) | 633 (485-781) | 1.26 | .249 | .404 |
| sHGFR | 254 (236-272) | 474 (87.6-860) | 1.13 | .308 | .491 |
| CXCL5 | 288 (216-361) | 340 (252-427) | 1.25 | .341 | .524 |
| sCathepsin D | 7.52 (5.44-9.60) | 9.10 (7.03-11.2) | 1.13 | .341 | .524 |
| sEMMPRIN | 387 (362-411) | 432 (354-509) | 1.12 | .376 | .548 |
| IL1RA | 12.1 (9.02-15.2) | 56.8 (0-147) | .84 | .376 | .548 |
| sIL6R | 92.5 (81.4-104) | 81.6 (64.6-98.6) | .94 | .376 | .548 |
| sCD30L | 4.26 (3.77-4.75) | 8.40 (2.25-14.6) | 1.08 | .452 | .626 |
| sHER3 | 228 (201-255) | 363 (166-559) | 1.09 | .452 | .626 |
| hGH | 601 (193-1009) | 948 (475-1420) | .82 | .452 | .626 |
| MMP3 | 1.39 (1.28-1.49) | 3.55 (0-7.86) | .96 | .458 | .626 |
| TNFα | 1.47 (1.47-1.47) | 1.46 (1.32-1.60) | 1.00 | .511 | .676 |
| sCD69 | 154 (96.9-210) | 233 (129-337) | .98 | .535 | .676 |
| CXCL13 | 187 (148-227) | 239 (181-297) | 1.02 | .535 | .676 |
| sE selectin | 24.9 (15.0-34.8) | 21.8 (13.8-29.8) | .97 | .535 | .676 |
| sEGFR | 19.2 (16.4-22.0) | 24.5 (11.8-37.2) | .91 | .535 | .676 |
| CCL21 | 194 (173-216) | 179 (149-209) | 1.02 | .579 | .721 |
| sCD40L | 1108 (734-1483) | 1260 (980-1539) | 1.03 | .624 | .767 |
| sEGF | 392 (219-564) | 462 (373-550) | 1.13 | .671 | .802 |
| sHER2/neu | 81.5 (61.7-101) | 90.4 (71.9-109) | 1.21 | .671 | .802 |
| CCL2/MCP1 | 2133 (1721-2544) | 2701 (1856-3546) | 1.12 | .720 | .848 |
| OPG | 1086 (940-1231) | 1208 (997-1420) | 1.19 | .769 | .894 |
| PSA | 4.32 (2.24-6.40) | 13.8 (0-30.0) | .57 | .796 | .913 |
| sHGF | 26.9 (22.8-31.1) | 26.9 (20.7-33.1) | .95 | .820 | .915 |
| sPECAM1 | 15.6 (12.6-18.7) | 17.3 (13.1-21.6) | .99 | .820 | .915 |
| sEpiregulin | 3.24 (2.89-3.59) | 4.67 (1.56-7.77) | 1.00 | .854 | .924 |
| Follistatin | 28.5 (24.8-32.2) | 28.8 (22.5-35.0) | 1.06 | .871 | .924 |
| IL8 | 215 (178-253) | 299 (159-440) | .89 | .871 | .924 |
| MPO | 49.4 (23.0-75.7) | 39.3 (29.4-49.1) | .98 | .871 | .924 |
| IL7 | 10.8 (8.86-12.8) | 10.9 (9.44-12.3) | .94 | .922 | .967 |
| suPAR | 425 (373-476) | 417 (337-497) | 1.04 | .974 | .997 |
| sVEGFR2 | 13.8 (12.5-15.0) | 29.9 (.11-59.7) | 1.01 | .974 | .997 |
| sHER4 | 174 (163-184) | 176 (143-208) | 1.02 | 1.000 | 1.000 |
| TNFSF14 | 22.0 (15.5-28.4) | 21.1 (17.0-25.2) | 1.06 | 1.000 | 1.000 |

FC (Fold Change) between group medians of linear ddCq

**P_corr_* value corrected for multiple comparisons (Benjamini-Hochberg correction)
